# Antibacterial potential of five phages in controlling *Enterococcus faecalis* and *Enterococcus faecium*

**DOI:** 10.1186/s12985-026-03147-9

**Published:** 2026-06-02

**Authors:** Henni Tuomala, Tiina Nylund, Markus Mustonen, Annika Flod, Saija Kiljunen

**Affiliations:** 1 Human Microbiome Research Program, Faculty of Medicine, University of Helsinki, Helsinki, Finland; 2 European Society of Clinical Microbiology and Infectious Diseases (ESCMID), Study Group for Non-traditional Antibacterial Therapy (ESGNTA), Basel, Switzerland

## Abstract

**Background:**

*Enterococcus faecium* and *Enterococcus faecalis* are opportunistic pathogens that form an increasing concern for hospital-acquired infections due to their natural and acquired antimicrobial resistance (AMR). Although phage therapy has gained global interest as a response to emerging AMR, *Enterococcus* remains an understudied target for phage therapy. In this context, we aimed to isolate *Enterococcus*-specific phages and study their in vitro infection efficacy when combined into cocktails, in the presence of antibiotics and human serum and against bacterial biofilms.

**Results:**

We isolated one *E. faecium*-phage fHoEfm07, that did not resemble any known phage genera and four *E. faecalis*-phages belonging to *Saphexavirus* (fHoEfa01 and fHoEfa06) and *Efquatrovirus* (fHoEfa03 and fHoEfa04) genera. The phages from the genus *Saphexavirus* and fHoEfm07 were suitable for therapeutic applications based on their genome characterization; however, the phages belonging to the *Efquatrovirus* genus contained a potential AMR-related gene. In vitro studies indicated that interactions between phages, antibiotics, and human serum depended on the specific phage-host pairing or antibiotic concentration. Moreover, only phage fHoEfa03 reduced the biofilm masses after 3 h and 24 h phage treatment.

**Conclusions:**

This study provides valuable insights into the potential of *Enterococcus* phages in conditions mimicking phage therapy. The characterization of novel phages, assessment of their therapeutic suitability, and exploration of synergistic treatment strategies contribute to the foundational knowledge required to advance phage therapy.

**Supplementary Information:**

The online version contains supplementary material available at 10.1186/s12985-026-03147-9.

## Background

*Enterococcus faecium* and *Enterococcus faecalis* are gram-positive bacteria that inhabit the environment, as well as the human and animal guts [1]. They are a significant cause of nosocomial infections through their intrinsic and acquired antimicrobial resistance (AMR) mechanisms and their role as reservoirs of AMR genes that can be transmitted to new bacterial cells [2]. Antimicrobial treatment options are scarce against enterococci, and the emergence of vancomycin-resistant *Enterococcus* (VRE) isolates further limit the available treatment options [3]. Phage therapy is re-emerging as a treatment for infections caused by AMR bacteria. Phage cocktails and phage-antibiotic combination therapies are promising treatment strategies as they enhance therapeutic outcomes by outcompeting bacterial resistance mechanisms [4]. For example, in one case study, *E. faecium*-phage cocktail eradicated a liver transplant-related VRE infection in a one-year-old patient, and in another, a patient with osteoarthritis of the left hip went through two phage cocktail-antibiotic treatment periods before eradication of the infective bacteria [5, 6]. The efficacy of phage therapy and phage-antibiotic combination treatments depends on the specific phage and antibiotic, which makes these interactions difficult to predict [7–11]. Phage-antibiotic synergy has been reported against various gram-positive and gram-negative bacterial species. For example, *Enterococcus* phages have been shown to act in synergy with vancomycin and daptomycin [9, 12].

The efficacy of antimicrobial treatments is further reduced by biofilms that can prevent the penetration of antibiotics and phages into the biofilm matrix. Biofilm formation also plays a key role in inducing severe enterococcal infections, as many *Enterococcus* virulence factors facilitate biofilm formation. Medical equipment like catheters and water infrastructure are common sites for enterococcal biofilm formation. Catheter-associated urinary tract infections (UTIs) are often chronic and difficult to treat, and in water infrastructures, biofilms release bacterial cells into the water, contributing to the development of waterborne infections [13, 14]. The available data suggest that phages and phage-antibiotic combination therapies are more successful in eradicating *Enterococcus* biofilms than antibiotic treatment alone [15]. The efficacy of phage treatment is further complicated by the infection environment, especially the human immune system, which can affect phage treatment efficacy in various ways. Antibody formation can reduce the number of viable phage particles in infection sites. Meanwhile, the complement system has been shown to act in synergy with phages against *Klebsiella pneumoniae* and *Escherichia coli* [16, 17].

*Enterococcus* phages remain understudied in comparison to other ESKAPEE bacteria such as *Staphylococcus aureus* or *K. pneumoniae*, despite their clinical significance [18]. In this work, we describe the characterization of five *Enterococcus* phages against clinical *E. faecium* and *E. faecalis* isolates. We studied the genomes of the phages and their host bacteria. In addition, we investigated the host specificity of the phages and their potency when combined as cocktails, as well as in conjunction with antibiotics and human serum. Finally, we evaluated their capacity to disrupt pre-formed biofilms.

## Materials and methods

### Bacterial strains, growth media, and basic phage methods

The bacterial strains used in this study were clinical *E. faecium* and *E. faecalis* strains (Supplementary Table S1). The strains were grown in lysogeny broth (LB) [19] or BD BactoTM Brain Infusion Broth (BHI) (Becton, Dickinson and Company, USA) that were supplemented with 1.5% agarose to prepare plates and 0.4% agarose to prepare soft agar. Phages were quantified using the standard double-layer plating method described by Sambrook et al. [19].

### Phage isolation and production

Wastewater samples were collected from Meilahti, Jorvi, and Peijas hospitals of the Helsinki and Uusimaa hospital district (HUS) (Finland) and were screened for phages against clinical *E. faecium* and *E. faecalis* isolates. For phage isolation, a mixture of wastewater (1.5 ml), growth media (4 ml), and host bacteria in the exponential growth phase was incubated overnight. Bacteria were removed with centrifugation (SL 16R, Thermo Fisher Scientific) at 4,000–5,000 g, 10–15 min, and filtering with 0.22 μm PES filters (Sartorius). Phages were detected with the standard double-layer plating method, and the obtained plaques went through three to five rounds of plaque purification [19]. Phages were produced for further assays as liquid cultures that were incubated for up to 5 h at 37 °C while shaking. Bacteria were removed as in phage isolation.

### Transmission electron microscopy

Phages were purified for imaging with the PEG precipitation method as described earlier [19, 20]. Before imaging, the buffer was changed to 0.1 M ammonium acetate and the sample was concentrated using Vivaspin 6 ultrafiltration units (100,000 MVCO, PES-membrane) (Sartorius, Helsinki, Finland). Phage titers were determined with the double-layer plating method. For imaging, 5 µl of purified phage lysate was added to a carbon-coated copper grid for 1 min. The grid was negatively stained for 1 min with 5 µl of 2% uranyl acetate and washed twice with 5 µl of mQ-H2O. The phages were imaged using JEOL JEM-1400 (Jeol Ltd., Tokyo, Japan) microscope under 80 kV, paired with a Gatan Orius SC 1000B bottom-mounted CCD camera (Gatan Inc., USA). The phage particle measurements were determined in Fiji-ImageJ program, version 1.54p (https://imagej.net/software/fiji/) by calculating the mean and standard deviation from ten individual phage particles for each phage.

### Phage genome isolation, sequencing, and analysis

The phage genomic DNA was isolated by treating the samples with 1 µg/µl of DNaseI and RNaseA for 30 min at 37 °C, followed by capsid degradation with Proteinase K (20 mg/ml) treatment at 56 °C for 1 h. The DNA was extracted using the Phage DNA Isolation (Norgen Biotek Corp.) kit or Viral TNA extraction kit with Promega Maxwell RSC device and was quantified using Qubit^TM^ Fluorometer device and Qubit^TM^ dsDNA BR assay kit (Thermo Fisher Scientific, USA) according to manufacturer’s instructions.

Phage genomic DNA (gDNA) was sequenced at Novogene (UK) on the Illumina NovaSeq 6000 platform, and phage genomes were assembled using the A5 pipeline (version: 0.7.5a-r405) [21]. Phage terminal ends were determined with PhageTerm (version 1.0.12) [22]. Prokka v1.14.6 was used for annotations that were verified with HHblits from HH-suite v3.3.0 against Phrogs v4, Cogs v2020, and Pvogs v2016 libraries using the Phagenomics platform (https://www.phagenomics.net). AMR genes and Virulence factors were identified with CARD and RAST, respectively [23–25]. Annotated phage genomes were submitted to GenBank with accession numbers presented in Table 1. Phylogenetic trees were constructed using ViPTree v4.0 from complete phage genomes with normalized tBLASTx scores (SG; 0 ≤ SG ≤ 1) above SG 0.63 [26] and were visualized in iTOL v7.1 [27]. The identities of the phages belonging to the same genus were determined using Blastn and were aligned with their closest relatives using DiGAlign software v2.0 [28].

**Table 1:** Details of the phage genomes

GenBank accession	fHoEfa01	fHoEfa03	fHoEfa04	fHoEfa06	fHoEfm07
	PV296017	PV296018	PV296019	PV296020	PV296021
Capsid length (nm)	101.80 ± 2.14	50.03 ± 2.11	53.0 ± 2.0	90.93 ± 4.78	54.61 ± 2.88
Capsid width (nm)	43.99 ± 2.28	51.35 ± 1.97	51.1 ± 1.23	39.96 ± 1.83	56.46 ± 2.35
Tail length (nm)	142.02 ± 6.04	194.70 ± 5.50	195.38 ± 4.51	128.40 ± 6.18	290.47 ± 7.61
Full length (nm)	243.82 ± 7.74	244.74 ± 5.87	248.37 ± 5.08	219.33 ± 10.5	345.07 ± 8.65
**Genome** **features**					
Genome size (bp)	56,771	41,011	41,154	56,951	44,400
GC-content	40.22%	34.75%	34.72%	40.15%	40.88%
Number of ORFs	93	64	66	94	60
Annotated proteins	41%	59%	48%	46%	45%
Hypothetical proteins	59%	41%	52%	54%	55%
tRNAs	1	0	0	1	0
**Predicted** **phage taxonomy**					
Class	*Caudoviricetes*	*Caudoviricetes*	*Caudoviricetes*	*Caudoviricetes*	*Caudoviricetes*
Family	Unclassified	Unclassified	Unclassified	Unclassified	Unclassified
Genus	*Saphexavirus*	*Efquatrovirus*	*Efquatrovirus*	*Saphexavirus*	Unclassified
**Host**	*E. faecalis* #6467	*E. faecalis* #6467	*E. faecalis* #6933	*E. faecalis* #7186	*E. faecium* #5864

Putative phage receptor binding proteins (RBP) of each phage genus were aligned with MAFFT alignment in Geneious Prime v2025.0.3 [29]. Approximately-maximum-likelihood phylogenetic trees were generated with FastTree tool v2.1 and visualized with iTOL v7.1 using proteins from complete phage genomes with the following Blastp inclusion criteria: fHoEfa01 (Identity: 89% and Coverage: 70%), fHoEfa03 (Identity: 75% and Coverage: 100%), fHoEfa04 (Identity: 78% and Coverage: 70%), fHoEfa06 (Identity: 97% and Coverage: 90%) and fHoEfm07 (Identity: % and Coverage: %) [27, 30]. All bioinformatic analyses were completed with standard settings.

### Isolation, sequencing and sequence analysis of bacterial strains

The gDNA of the phage isolation hosts was extracted for sequence analysis. The gDNA was isolated using the NucleoSpin^®^ Microbial DNA kit for microorganisms (Macherey-Nagel, Biotop) according to the manufacturer’s instructions. DNA concentrations were quantified with Qubit™ dsDNA BR assay kit (Thermo Fisher Scientific).

Sequencing was performed at Novogene UK on the Illumina platform. For genome assembly, we used the Comprehensive Genome Analysis tool (BV-BRC) with the genus *Enterococcus* and taxonomy ID 1350 [31]. Automatic annotation and detection of virulence-related genes were performed using the Rapid Annotation using Subsystem Technology (RAST) tool [25, 32, 33]. The sequence types were determined using MLST tool in Galaxy platform v2.22.0 against the PubMLST database. Prophages were identified with the Phage Search Tool Enhanced Release (PHASTER) tool, AMR genes were identified with the Resistance Gene Identifier (RGI) tool and the Comprehensive Antibiotic Resistance Database (CARD), and phage defense mechanisms were detected with the DefenceFinder tool by MDM labs, Paris [23, 24, 34, 35]. Standard settings were used for all bioinformatics analyses unless otherwise stated. The raw reads were submitted to the Sequence Read Archive (SRA) database under BioProject ID: PRJNA1355155.

### Proteomics analysis of fHoEfm07

A proteome analysis was performed for the phage fHoEfm07 as it did not resemble any earlier, characterized phages. fHoEfm07 was purified for the proteomics analysis with Vivaspin ultrafiltration units (Sartorius, Helsinki, Finland) and ion exchange chromatography (IEC). First, Vivaspin 20 (100,000 MVCO, PES-membrane) units were used to wash the samples twice with SM-buffer (100 mM NaCl, 10 mM MgSO4, 50 mM Tris-HCl, pH 7.5) for buffer exchange and concentrate the sample. The pre-washed phage lysate was then purified using an ÄKTA FPLC device (GE Healthcare, Helsinki, Finland) paired with a 1 ml CIMmultus QA anion exchange column (Sartorius BIA Separations, Ajdovščina, Slovenia). The sample was injected into the column, and the unbound proteins were washed with 8 column volumes (CV) of Buffer A (20 mM Tris-HCl, pH 7.5). Lightly bound proteins were washed with 6 CV of Buffer A supplemented with 110 mM NaCl, and the sample was eluted with 8 CV of Buffer A supplemented with 200 mM NaCl. Fractions containing the phage were collected and pooled, and the elution buffer was changed to SM buffer with the Vivaspin 6 ultrafiltration units (100,000 MVCO, PES-membrane). The final volume was adjusted to 350 µl.

The phosphoproteomics analysis was conducted at the Proteomics unit (Institute of Biotechnology, University of Helsinki, Helsinki, Finland) using their in-house methodology. First, the sample was phosphoenriched and proteolytically digested into peptides. The peptides were desalted and analysed on LC-MS/MS. A data-independent acquisition (DIA) mode was used for data acquisition and the data was analyzed using the DIA-NN software (www.github.com/vdemichev/diann), which allows for peptidoform-confident protein identification. The obtained data was analyzed by comparison against the annotated amino acid sequences of phage fHoEfm07 and its host strain #5864. The number of proteolytic sequences cut-off value 3 was used for protein identification.

### Phage host range determination

The phage host ranges were determined in 51 *E. faecium* and *E. faecalis* strains (Supplementary Table S1) with a modified liquid assay described by Patpatia et al. [30]. For the assay, 200 µl of 1:50 diluted overnight bacterial culture was mixed with 10 µl of phage lysate (10^8^ PFU/ml) or BHI broth [30]. The bacteria were grown at 37 °C while shaking and the growth was measured at OD_600_ at 20- or 30-min intervals using the optical density reader Logphase 600 (Biotek) or Bioscreen FP-1100-C (Oy Growth Curves Ab Ltd, Finland). Phage with its original host was used as a positive control, and BHI broth was used as a negative control. The assay time was extended to 10 h to ensure bacterial growth. All samples were tested in triplicate, which were used to calculate mean values and standard deviation. A strain was considered susceptible when the OD_600_-values were < 50% of the bacterial control, intermediate when OD_600_-values were 50–85%, and resistant when OD_600_-values were > 85% at the 7 h timepoint. A standard double-layer spot assay was used to test those strains that did not grow during the liquid assay, using 10 µl drops of a serial dilution of phage lysates. LB broth (10 µl) was used as a negative control [19]. All calculations and graphical illustrations were performed in Origin 2024b.

### Efficacy of phage cocktails

The interactions between the isolated *E. faecalis* phages were studied as cocktails of two or three phages. Table 2 presents the cocktail combinations and the test strains for each cocktail. All possible combinations of two and three phages were tested against the strains that were infected by all phages included in a cocktail. The cocktails contained an equal amount of each phage to reach a total of 10^8^ PFU/ml. The cocktail killing efficacies were studied using the standard liquid assay. The bacterial optical density was followed for 24 h and was measured at 30 min intervals.

**Table 2:** *Enterococcus* phage cocktails and test strains

Cocktail	#6467	#6933	#7186	#5722	#6934
fHoEfa01	x		x	x	
fHoEfa03	x		x	x	
fHoEfa04		x		x	x
fHoEfa06	x		x	x	x
fHoEfa01 + fHoEfa03	x		x	x	
fHoEfa01 + fHoEfa04				x	
fHoEfa01 + fHoEfa06	x		x	x	
fHoEfa03 + fHoEfa04				x	
fHoEfa03 + fHoEfa06	x		x	x	
fHoEfa04 + fHoEfa06				x	x
fHoEfa01 + fHoEfa03 + fHoEfa04				x	
fHoEfa01 + fHoEfa03 + fHoEfa06	x		x	x	
fHoEfa01 + fHoEfa04 + fHoEfa06				x	
fHoEfa03 + fHoEfa04 + fHoEfa06				x	

x indicates that phage or cocktail was tested in the respective strain

### Phage-antibiotic interaction assay

Before phage-antibiotic synergy testing, the antibiotic susceptibilities of the bacterial strains were first determined by the liquid culture assay in the presence of 200 µg/ml, 150 µg/ml, 100 µg/ml, 50 µg/ml, 30 µg/ml, 10 µg/ml, 5 µg/ml, and 2.5 µg/ml of vancomycin, 80 µg/ml, 60 µg/ml, 40 µg/ml, 20 µg/ml, 10 µg/ml of daptomycin, 400 µg/ml, 300 µg/ml, 200 µg/ml, 100 µg/ml, 50 µg/ml and 10 µg/ml of ampicillin, and 800 µg/ml, 600 µg/ml, 400 µg/ml, 200 µg/ml, 100 µg/ml and 10 µg/ml of piperacillin. *E. faecium* strains were tested with vancomycin and daptomycin, and *E. faecalis* strains with ampicillin, piperacillin, and vancomycin according to the treatment practices used in hospitals of the Hospital district of Helsinki and Uusimaa (HUS) at the time of the study.

Phage-antibiotic combinations were tested with fHoEfm07 with the liquid assay method in three antibiotic concentrations representing high, medium, and low-test concentrations: 80 µg/ml, 20 µg/ml and 10 µg/ml for daptomycin and 200 µg/ml, 50 µg/ml, and 5 µg/ml for vancomycin.

### Phage-serum interaction assay

The phage – human serum assay was used to study the phage infection efficacy in the presence of commercial normal human serum. The assay was performed with modified liquid assay; 1:50 dilution of *Enterococcus* host or 1:500 dilution of serum-sensitive *E. coli* strain DH10B was prepared in BHI mixed with 1:1 ratio of Tris++ buffer (10 mM Tris-HCl, 150 mM NaCl, 2 mM CaCl_2_, 0.5 mM MgCl_2_, pH = 7.3) [31]. The bacterial dilution was combined with phage (10^8^ PFU/ml) in 10 µl diluted in BHI or BHI as non-phage control, and a final concentration of 30%, 10% and 3% of normal human serum (NHS, Human serum H4522, Sigma-Aldrich, USA), heat inactivated human serum (HIS) or BHI to reach final volume of 200 µl. HIS was prepared by incubating NHS at 56 °C for 1 h. The bacterial growth was followed for 24 h, and the OD_600_ was measured at 30 min intervals.

### Biofilm assay

The capacity of the phage to degrade preformed bacterial biofilms was studied with a method adapted from Goudani et al. [32, 33]. A 10^3^-fold dilution of an overnight bacterial culture, grown in BHI broth, was incubated at 37 °C and 180 rpm for 4 h. On a 96-well plate, 160 µl of bacteria (10^7^ CFU) or BHI was added, and the biofilms were allowed to form at 37 °C + 5% CO_2_ for 24 h. The supernatants were replaced with 160 µl of phage (10^6^ PFU) in BHI or BHI and incubated for 3–24 h at 37 °C + 5% CO_2_. The planktonic cell counts were determined from the supernatants by plating 100 µl of a serial dilution and incubating at 37 °C + 5% CO_2_ overnight. For biofilm quantification, biofilms were washed thrice with phosphate-saline buffer (PBS), dried for 30 min, and stained with crystal violet (0.2%) for 10 min. The excessive stain was washed thrice with water and the remaining stain was dissolved in 33% acetic acid. For quantification, 1:10 diluted samples were measured at A_595nm_ (Hidex Sense plate reader, Hidex Oy, Finland). Three biological replicates were prepared of each sample and control. The background signal of A_595nm_ measurement was removed and these were used to calculate the mean values and standard deviation. Paired Sample t-test was used for statistical testing with 0.05 confidence level.

## Results

### Phages were isolated from sewage water against clinical *E. faecalis* and *E. faecium* strains

**Fig. 1:**
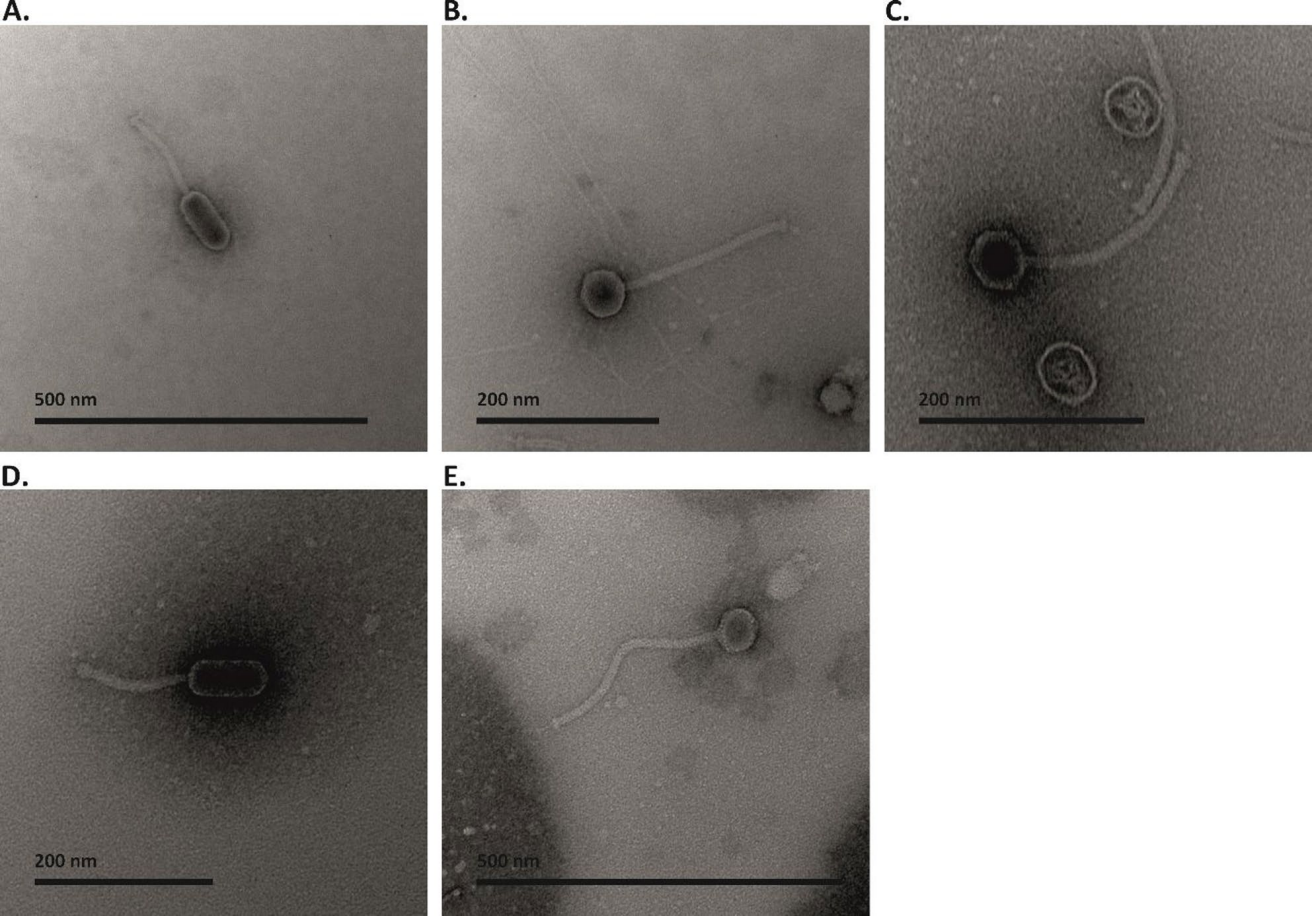
Negatively stained phage phages imaged with Transmission electron microscopy (TEM). **A**. fHoEfa01 **B**. fHoEfa03 **C**. fHoEfa04 **D**. fHoEfa06 E. fHoEfm07. Bars represent 500 nm in images **A**. and **E**. and 200 nm in images **B-D**.

One *E. faecium*-specific phage, fHoEfm07, and four *E. faecalis*-specific phages, fHoEfa01, fHoEfa03, fHoEfa04, and fHoEfa06, were isolated from hospital wastewater mixture against clinical *Enterococcus* isolates. TEM imaging revealed that all of the isolated phages had a siphovirus morphology. The *E. faecalis* specific phage particles were 219–248 nm long, while the length of fHoEfm07 was 345 nm (Fig. 1A-E). The precise measurements and standard deviation values are presented in Table 1. Clear differences were seen in the capsid shapes and tail lengths. fHoEfa01 and fHoEfa06 had elongated capsids and shorter tails than the other phages (Fig. 1A and D). fHoEfa03, fHoEfa04 and fHoEfm07 had icosahedral capsids (Fig. 1B, C and E) and a longer tail than fHoEfa01 and fHoEfa06. Of note, the tail of fHoEfm07 was longer than the tails of the other phages, although its capsid was approximately the same size as the ones of fHoEfa03 and fHoEfa04 (Fig. 1E).

The phage taxonomies, genome size, GC-content, number of putative open reading frames (ORFs), % of hypothetical proteins, GenBank accession numbers and host strains are presented in Table 1. The PhageTerm analysis identified for fHoEfa01 and fHoEfa06 a Headful packaging mechanism and COS (3’) packaging mechanism for the fHoEfa03 and fHoEfa04. However, PhageTerm did not identify the packaging mechanism of fHoEfm07, and its genome was organized to start from the small terminase subunit as recommended by Turner et al. [34].

**Fig. 2:**
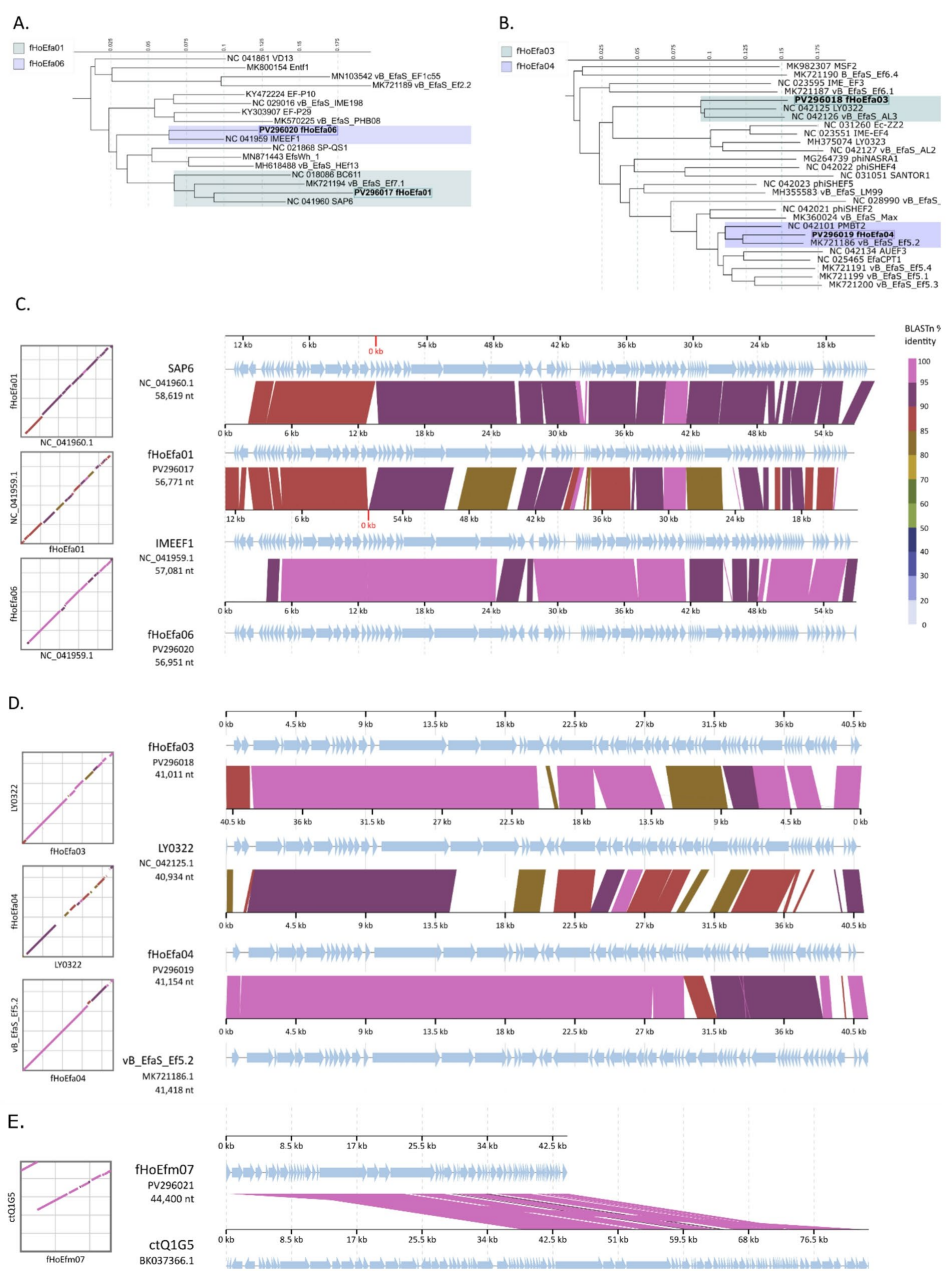
Phylogenetic trees and whole genome alignments of the isolated phages. **A**. Proteomic tree of fHoEfa01 and fHoEfa06 with related complete phage genomes. **B**. Proteomic tree of fHoEfa03 and fHoEfa04 with related complete phage genomes. **C**. Whole-genome alignment of fHoEfa01 and fHoEfa06 with their closest relatives. **D**. Whole-genome alignment of fHoEfa03 and fHoEfa04 with their closest relatives. **E**. Whole-genome alignment of fHoEfm07 with its closest relative. Phages that had normalized tBLASTx scores (SG; 0 ≤ SG ≤ 1) above SG 0.63 were included in the phylogenetic analysis. The trees were generated with ViPTree v4.0 and visualized in iTOL v7.1, and the phages isolated in this work are highlighted. DiGAlign v2.0 was used for whole-genome alignments. The red line indicates the original starting point of the genome that were rearranged for alignment.

Phylogenetic analysis classified phages fHoEfa01 and fHoEfa06 to the *Saphexavirus* genus and phages fHoEfa03 and fHoEfa04 to the *Efquatrovirus* genus (Figure 2A and B). fHoEfa01 and fHoEfa06 shared 88.9% sequence identity and the fHoEfa03 and fHoEfa04 shared 92.4% sequence identity. Phylogenetic trees were constructed from phage genomes with SG >0.63 and show that the closest relatives were SAP6 (NC_041960) for fHoEfa01 (SG 0.86), AL3 (NC_042126) for fHoEfa03 (SG 0.87), Ef5.2 (MK721186) for fHoEfa04 (SG 0.91) and IMEEF1 (NC_041959) for fHoEfa06 (SG 0.90). Figures 2C and D show the alignments of fHoEfa01 and fHoEfa06, and fHoEfa03 and fHoEfa04 with their closest relatives. fHoEfm07 differed from known, classified *Enterococcus* phages but showed similarity to a partial metagenomic sequence, ctQ1G5 (GenBank accession: BK037366.1) of human origin [36]. Thus, a phylogenetic tree was not constructed from fHoEfm07, but its genome was aligned with the ctQ1G5 sequence (GenBank: BK037366.1) and shown in Figure 2E. fHoEfm07 showed similarity only against the end of the reference genome, while the beginning of ctQ1G5 was similar to other partial phage genomes from metagenome assemblies (not shown).

The annotated ORFs encoded for structural, replication, DNA manipulation and packaging, and host lysis-associated proteins. Known virulence factors or known genes indicating lysogeny were not found in the phage genomes, and fHoEfa01, fHoEfa06 and fHoEfm07 were free of known AMR genes. However, fHoEfa03 and fHoEfa04 carried a putative AMR-related gene encoding a metal-dependent hydrolase.

**Fig. 3:**
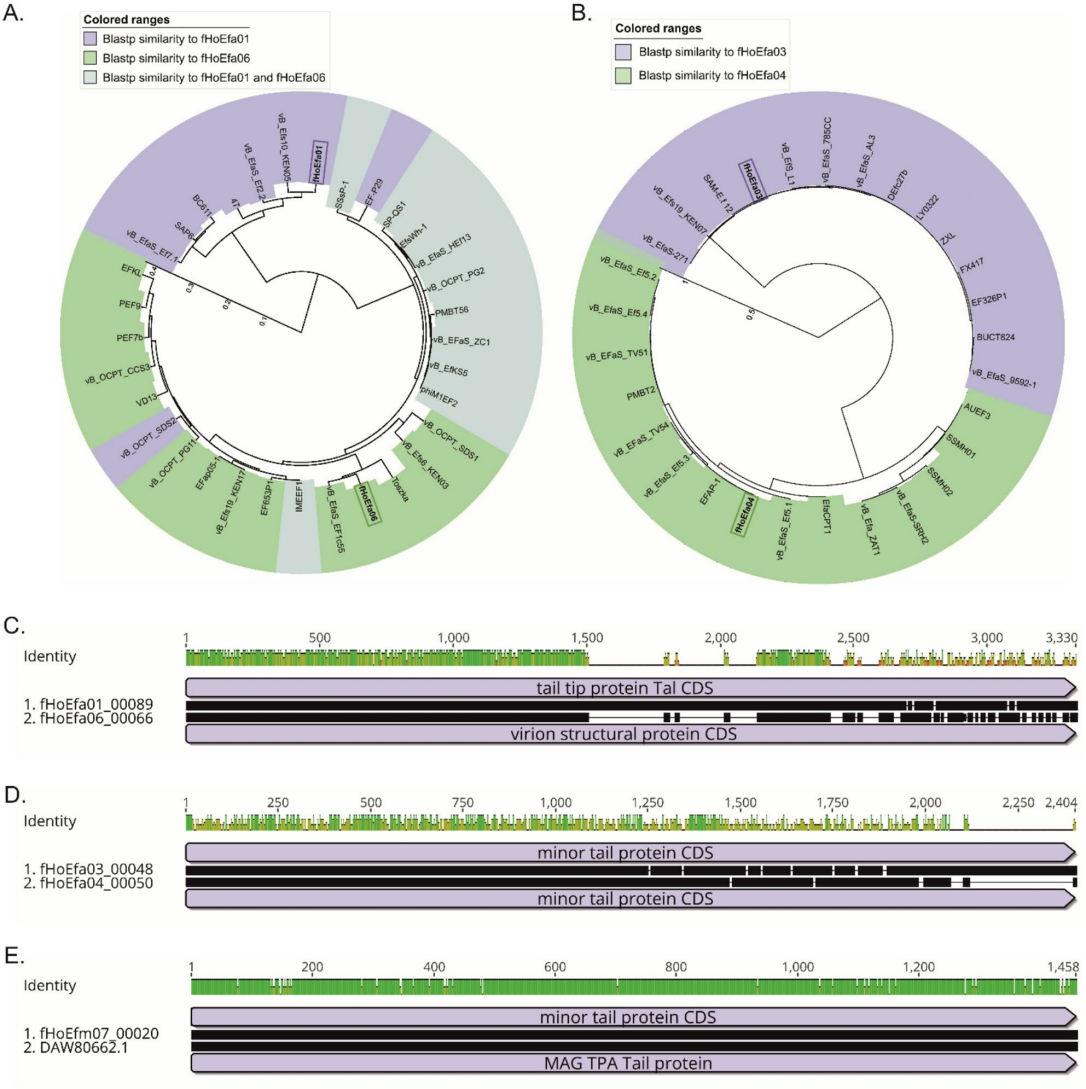
Phylogenetic analysis and alignment of phage tail endopeptidases. **A**. Approximately-maximum-likelihood phylogenetic tree of fHoEfa01 and fHoEfa06 tail endopeptidases. The fHoEfa01 and fHoEfa06 proteins are highlighted. **B**. Approximately-maximum-likelihood phylogenetic tree of fHoEfa03 and fHoEfa04 tail endopeptidases. The fHoEfa03 and fHoEfa04 are highlighted. **C**. Alignment of fHoefa01 and fHoEfa06 tail endopeptidases. **D**. Alignment of fHoEfa03 and fHoEfa04 tail endopeptidases. **E**. fHoEfm07 tail endopeptidase aligned with MAG TPA Tail protein from an unclassified *Caudoviricetes*. The phylogenetic trees were created with FastTree v2.1 and visualized with iTOL v7.1. The alignments were performed and visualized with the MAFFT tool in Geneious Prime v2025.0.3. Green color indicates 100% sequence identity, while yellow color indicates >30% identity and red <30% identity.

We identified the putative receptor binding proteins (RBPs) from all phages (fHoEfa01_00089, fHoEfa03_00048, fHoEfa04_00050, fHoEfa06_00066 and fHoEfm07_00020). All these proteins contained a phage endopeptidase domain (Pfam: PF06605) and belonged to the group of phage tail endopeptidases. The most similar proteins to the endopeptidases of each phage genus are shown in the phylogenetic trees (Fig. 3A and B). Endopeptidases from fHoEfa01 and fHoEfa06 shared 94.3% amino acid identity with 55% coverage, while the endopeptidases of the fHoEfa03 and fHoEfa04 shared 56.8% amino acid identity and 64% coverage (Fig. 3C and D). The N-terminal end, which is attached to the phage, of the fHoEfa01 and fHoEfa06 endopeptidases showed most similarity. Interestingly, the host receptor binding part, C-terminal end, of the endopeptidases differed between the phages, indicating that they may use different receptors (Fig. 3C). In contrast, the sequence identity of fHoEfa03 and fHoEfa04 endopeptidases differed throughout the sequence (Fig. 3D). The endopeptidase of fHoEfm07 resembled only one tail protein from an unclassified *Caudoviricetes* and their alignment is shown in Fig. 3E.

### Genomic features of bacterial strains used for phage isolation

The genomes of the isolation hosts of the isolated phages were characterized to study their suitability for phage propagation for therapeutic applications. The MLST typing showed that the host strains #6467 and #7186 belonged to ST16, while strain #6933 belonged to ST 81 and #5864 to ST 2026. None of the host genomes contained known toxin genes. However, nine and ten genes that were associated with invasion and intracellular resistance, were present in the *E. faecalis* strains and the *E. faecium* strain, respectively (Supplementary Table S2). Interestingly, these genes were annotated as Mycobacterial genes, probably because they share functional and/or structural similarities to genes found in *Mycobacterium*. In addition, all the host strains harbored prophages, AMR-related genes, and phage defense mechanisms. One incomplete prophage was found in strains #6933 and #5864, while host #6467 carried one incomplete and one intact prophage (Supplementary Table S3). In contrast, the host #7186 harbored a total of five prophage genomes, of which two were intact and three were incomplete (Supplementary Table S3). All of the *E. faecalis* strains contained the incomplete prophage PHAGE_Entero_vB_IME197_NC_028671 [7] (Supplementary Table S3). One intact prophage region in host #7186 (PHAGE_Entero_EFC_1_NC_025453 [41]) included a putative virulence associated gene, an ImmA/IrrE family metallo-endopeptidase, which is part of the toxin-antitoxin ToxN/AbiQ system found in *Geobacillus* [37]. However, even though *Geobacillus* belongs to the same order as *Enterococcus*, it is not known if the ImmA/IrrE gene has a toxic function in *Enterococcus*, as RAST did not detect this gene as a virulence-related gene. Other prophage regions in the *E. faecalis* and *E. faecium* strains did not contain genes associated with virulence. Between two and ten AMR-associated genes were found in all *Enterococcus* genomes (Supplementary Table S4). Host #6467 carried most AMR-associated genes (*n* = 10), and the found genes were predicted to be associated with antibiotics from 12 different drug classes through multiple mechanisms (Supplementary Table S4). The AMR genes in hosts #6933 and #7186 were predicted to be associated with antibiotics from five and six different classes, respectively, while the two AMR genes in #5864 were involved with glycopeptide antibiotics and aminoglycosides (Supplementary Table S4). For phage defense mechanisms, all four *E. faecalis* strains carried Type I and Type II restriction-modification (r-m) systems and the RosmerTA toxin-antitoxin system. *E. faecium* strain #5864 harbored the type IV r-m system and CRISPR/Cas system with Cas Class 2 subtype II A 1 protein.

### Proteomics analysis of fHoEfm07

The proteome analysis was performed for phage fHoEfm07 to identify the structural proteins of the phage, as fHoEfm07 resembles only one, earlier phage from a metagenome assembly. The LC-MS/MS analysis detected a total of 12 proteins that originated from the fHoEfm07 genome and are presented in the Supplementary Table S5. The identified proteins included nine structural proteins, two DNA binding proteins, and one hypothetical protein (Supplementary Table S5)

### *E. faecalis*-specific phages were species-specific, unlike the *E. faecium* phage fHo-Efm07

A set of 51 clinical *E. faecium* and *E. faecalis* strains was used to evaluate the host range and species specificity of the isolated phages. The assay showed that the *E. faecalis* phages were species-specific (Supplementary Table S1). fHoEfa01 and fHoEfa03 infected 5/51 (10%) and 3/51 (6%) of test isolates, respectively, while fHoEfa04 and fHoEfa06 had broader host ranges that covered 12/51 (24%) and 13/51 (25%) of the isolates, respectively. Furthermore, only fHoEfm07 infected both *E. faecalis* and *E. faecium* strains, although its host range was narrow and covered 3/51 (6%) of the test strains. Of the fHoEfm07-susceptible strains, two were *E. faecium* isolates and one was an *E. faecalis* isolate, which was intermediately susceptible to fHoEfm07.

### The phage-phage interactions varied depending on the cocktail composition and target strain

**Fig. 4:**
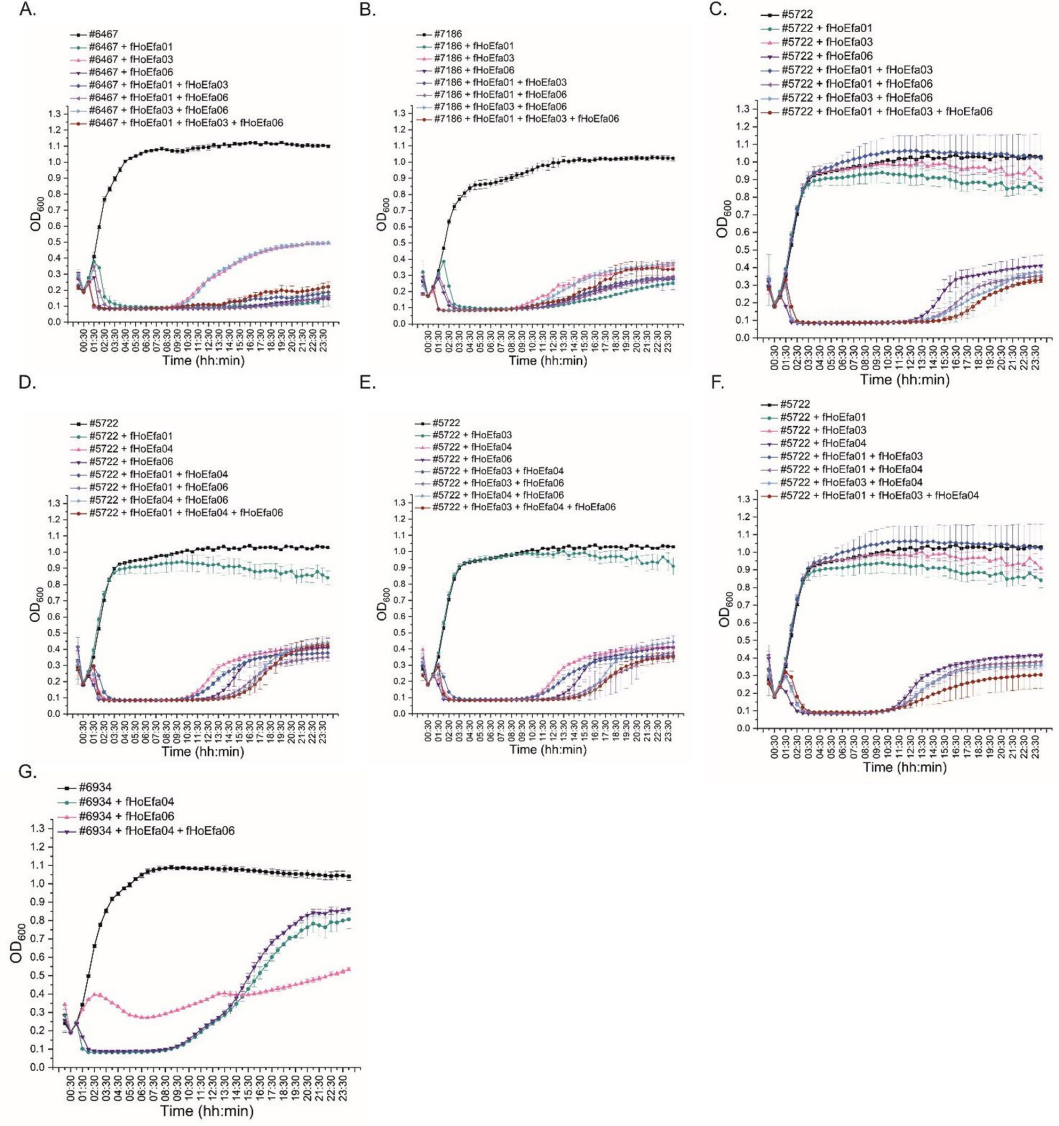
Phage cocktail efficacy assay. The efficacy of the *E. faecalis*-phages (fHoEfa01, fHoEfa03, fHoEfa04 and fHoEfa06) as cocktails was studied using the liquid assay, where the bacterial growth alone and in the presence of one to three phages was measured at OD_600_ for 24 h. BHI broth was used as a negative control and each sample was prepared in triplicate, which were used to calculate mean values and standard deviations. **A**. Phage cocktails against host #6467. **B**. Phage cocktails against host #7186. **C-F**. Phage cocktails against host #5722. **G**. Phage cocktails against host #6934. Origin 2024b was used for calculations and visualization

To study the potential of the *E. faecalis* phages for therapeutic applications, we evaluated their phage-phage interactions as part of two or three phage cocktails (Fig. 4). The strain #6467 was tested against fHoEfa01, fHoEfa03 and fHoEfa06 and their all possible two and three phage combinations (Fig. 4A). Out of the three phages, fHoEfa03 infected the strain less efficiently than the other two. In phage cocktails, fHoEfa01-fHoEfa03, fHoEfa01-fHoEfa06 and fHoEfa01-fHoEfa03-fHoEfa06 combinations infected the strain #6467 as well as fHoEfa01 or fHoEfa06 alone. In contrast, the cocktail fHoEfa03-fHoEfa06 was as efficient as fHoEfa03 alone, and thus weaker than fHoEfa06 alone (Fig. 4A). The strain #7186 was also tested against the same phages (Fig. 4B). Here, fHoEfa01 alone was the most efficient when compared to the other single phages and phage combinations, although the difference to fHoEfa06 and the two-phage cocktails including fHoEfa01 was marginal. All four *E. faecalis*-specific phages and their two- and three-phage combinations were tested against strain #5722 (Fig. 4C-F). fHoEfa01 and fHoEfa03 infection was weak in strain #5722, and as a two-phage cocktail, their infection efficacy was further decreased to the level of no-phage control (Fig. 4C). Both fHoEfa04 and fHoEfa06 infected strain #5722 more efficiently, and all two and three-phage combinations containing either of these were at least slightly more efficient than any of the single phages. Furthermore, strain #6934 was tested against fHoEfa04 and fHoEfa06, in which their combination showed a weak inhibitory effect (Fig. 4G). This finding was different to strain #5722, where the same phage combination improved the infection efficacy compared to individual phages (Fig. 4D and E).

### fHoEfm07 showed synergistic effects with vancomycin and daptomycin

**Fig. 5:**
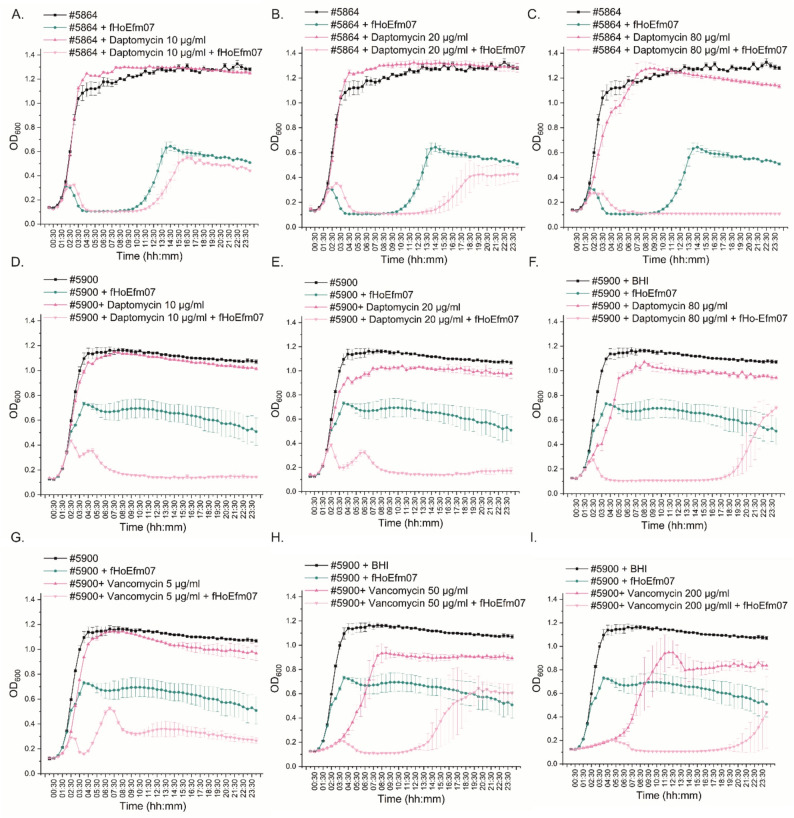
Phage-antibiotic interaction assay. The interactions between phage fHoEfm07 and three daptomycin and vancomycin concentrations were studied in *E. faecium* strains #5864 and #5900 using a liquid assay. The bacterial growth alone was compared to the bacterial growth in the presence of phage and/or antibiotic and BHI broth was used as a negative control. The bacterial growth was measured at OD_600_ for 24 h. Each sample was prepared as a triplicate, which were used to calculate the mean values and standard deviations. **A-C**. fHoEfm07 interactions with 10 µg/ml, 20 µg/ml and 80 µg/ml daptomycin against host #5864. **D-F**. fHoEfm07 interactions with 10 µg/ml, 20 µg/ml and 80 µg/ml daptomycin against host #5900. **G-I**. fHoEfm07 interactions with 5 µg/ml, 50 µg/ml and 200 µg/ml vancomycin against host #5900. Origin 2024b was used for calculations and visualization.

The phage interactions with antibiotics were studied to evaluate the suitability of phages for phage-antibiotic combination treatments. The preliminary antibiotic susceptibility assay showed that all *E. faecalis* strains used in this study were extremely susceptible to the tested antibiotics (not shown) and therefore were not tested in the phage combination studies. The *E. faecium* strain #5864 was susceptible to the tested vancomycin concentrations (not shown) but resistant to the tested daptomycin concentrations (Fig. 5A-C), and thus in this strain, only phage-daptomycin combination was tested. *E. faecium* #5900 was resistant to the tested daptomycin concentrations and vancomycin (Fig. 5D-I), and fHoEfm07 combinations with both antibiotics were tested in this strain.

fHoEfm07 showed variable interactions in combination with both tested antibiotics, as the bacterial killing efficacies were different in different antibiotic concentrations. Increasing the daptomycin concentration improved the combined killing efficacy against strain #5864 (Fig. 5A-C). In contrast, the effect of the phage-daptomycin treatment against strain #5900 was weakest with the highest daptomycin test concentration (80 µg/ml), the medium and low concentrations (20 µg/ml and 10 µg/ml, respectively) behaving similarly (Fig. 5D-F). The presence of vancomycin led to a very different fHoEfm07 behavior in comparison to its behavior in the presence of daptomycin. Here, fHoEfm07-vancomycin treatments were most efficient with the highest (200 µg/ml) and lowest (5 µg/ml) vancomycin combinations and weakest in the fHoEfm07-vancomycin (50 µg/ml) treated samples (Fig. 5G-I).

### The phage-host interactions were diverse in the presence of human serum

Phage-host interaction assay in the presence of human serum was used to evaluate the phage efficacy under conditions that mimic intravenous (iv) phage application. The effect of NHS and HIS on the bacterial strains is presented in Supplementary Figure S1. All strains were able to grow in the presence of NHS and HIS, however, there were slight differences between the strains. The presence of 30% NHS and HIS reduced to various degrees the growth of all *E. faecalis* strains, while 10% NHS reduced slightly the growth of #6933 and #7186 (Fig. S1A-I). Strain #7186 was the only strain slightly affected by 10% HIS and 3% NHS and HIS (Fig. S1 J-L). The *E. faecium* strain #5864 was resistant to all NHS and HIS concentrations (Fig. S1M-O).

The behavior of the phages in the presence of serum varied depending on the phage, serum concentration, and whether the serum was inactivated. All NHS and HIS concentrations inhibited fHoEfa01 infection, and the strongest inhibitory effect was observed in the presence of 30% NHS (Supplementary Fig. S1A-C). Meanwhile, fHoEfa03 infection improved in the presence of 30% NHS and was inhibited slightly in all other test conditions (Fig. 1D-F). fHoEfa04 infection efficacy was improved in the presence of all NHS and HIS concentrations, 30% NHS improving it the most (Fig. S1G-I). fHoEfa06 infection was slightly inhibited by the presence of 3% and 10% NHS and HIS. However, 30% NHS improved fHoEfa06 infection efficacy while 30% HIS had no effect (Fig. S1J-L). fHoEfm07 infectivity differed depending on the serum being NHS or HIS. The presence of 3% NHS did not affect fHoEfm07 infectivity, while 10% NHS delayed bacterial growth, and 30% NHS had no effect on the phage infection during the first four hours but resulted in lower OD_600_ -values at later time points. fHoEfm07 in the presence of 3% and 30% HIS delayed bacterial growth and/or lowered maximum OD_600_, while 10% HIS barely affected the phage infection (Fig. S1M-O).

### Phages had diverse capacities to reduce preformed biofilms

**Fig. 6:**
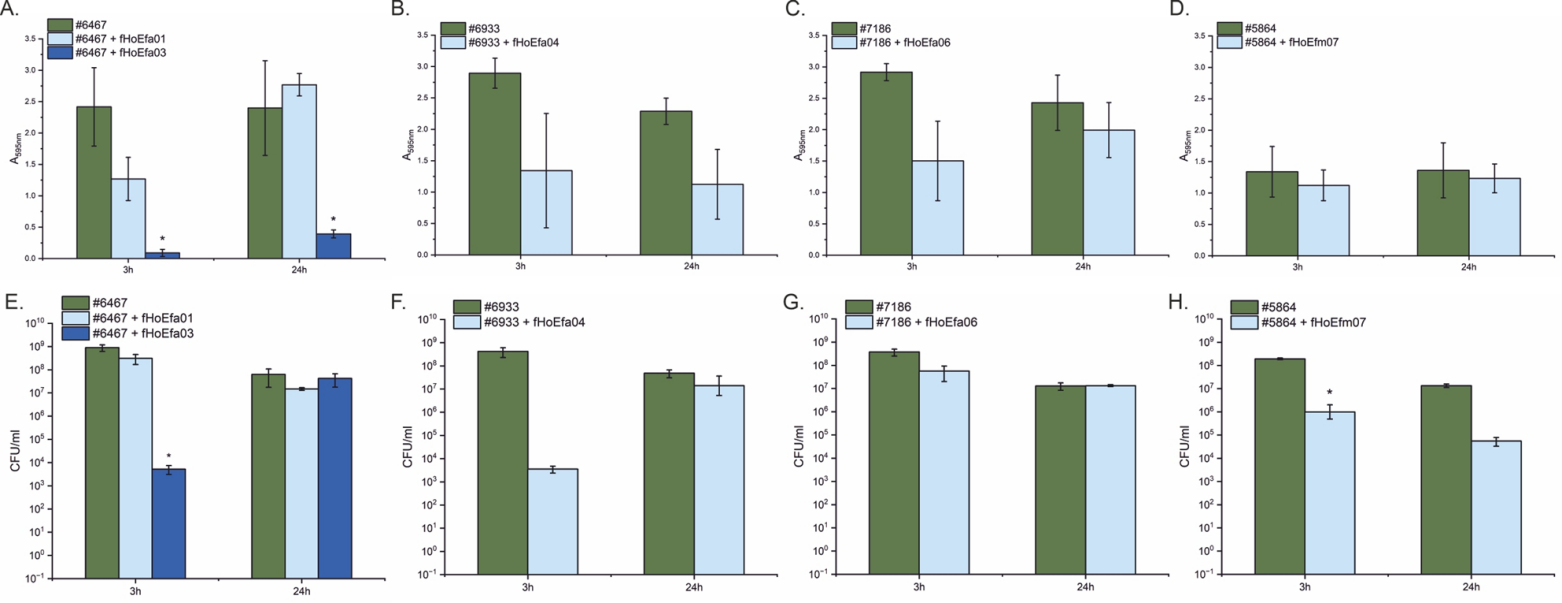
Biofilm assay. The phage efficacies against 24 h old bacterial biofilms were studied on polystyrene micro-well plates and crystal violet staining. The biofilms were quantified with A_590nm_ measurement, and the planktonic cell counts (CFU/ml) were determined by comparing phage-treated samples to untreated samples. Both measurements were repeated thrice and were used to calculate the mean values and standard deviations. **A**. fHoEfa01 and fHoEfa03 efficacy in degrading bacterial biofilms. **B**. fHoEfa04 efficacy in degrading bacterial biofilms. **C**. fHoEfa06 efficacy in degrading bacterial biofilms. **D**. fHoEfm07 efficacy in degrading bacterial biofilms. **E**. Planktonic cell counts of samples treated with phages fHoEfa01 and fHoEfa03. **F**. Planktonic cell counts of samples treated with phages fHoEfa04. **G**. Planktonic cell counts of samples treated with phages fHoEfa06. **H**. Planktonic cell counts of samples treated with phages fHoEfm07. Origin 2024b was used for calculations and visualization. Paired sample t-test was used for statistical analysis with a 0.05 confidence level. Significant difference was indicated with * symbol

To evaluate the biofilm degrading activity of the phages, the phage efficacy was tested against 24 h old biofilms with 3 h and 24 h phage incubation times. Reduction in biofilm masses were observed after 3 h treatment with phages fHoEfa01, fHoEfa03, fHoEfa04, and fHoEfa06, even though the reduction was statistically significant only for fHoEfa03, probably due to high standard deviation values. At 24 h timepoint statistically significant reduction was observed only with fHoEfa03, while statistically insignificant reduction was observed with fHoEfa04 and fHoEfa01 treatments (Fig 6A-C). fHoEfa06 did not reduce the bacterial biofilm mass at 24 h (Fig 6C) and fHoEfm07 during either incubation time (Fig 6D). Statistically significant reduction in planktonic cell counts were observed after 3 h treatment only with fHoEfa03 and fHoEfm07 phages (Fig 6E and H). Meanwhile, fHoEfa04 reduced the planktonic cell counts at 3 h timepoint, but the p-value between the treated and untreated samples was 0.061, just above the confidence level (*p*=0.05) (Fig 6F). At 24 h time point, only fHoEfm07 was able to reduce the planktonic cell counts, even though the reduction was not statistically significant (Fig 6H).

We also screened the phage genomes for genes associated with biofilm eradication. Each phage contained one endolysin and endopeptidase gene that has potential anti-biofilm activity, but no tail-associated depolymerases were identified. Conserved domains of the endolysins were shared between phages from the same genus (not shown), but the endopeptidases differed between the phages. These enzymes could explain the differences in anti-biofilm activity of the phages.

## Discussion

Enterococci have emerged recently as a significant cause of nosocomial infections and, therefore, remain an understudied target pathogen for phage therapy [18]. In this work, we characterized five novel phages, fHoEfa01, fHoEfa03, fHoEfa04, fHoEfa06 and fHoEfm07, from Finnish hospital wastewater against clinical *E. faecium* and *E. faecalis* isolates. The *E. faecalis* -phages belonged to *Saphexavirus* (fHoEfa01 and fHoEfa06) and *Efquatrovirus* (fHoEfa03 and fHoEfa04) genera, both known to include lytic phages. Phages that belong to the *Saphexavirus* and *Efquatrovirus* genera have a siphovirus morphology and medium sized genomes [37]. *Enterococcus* phages with a siphovirus morphology can be classified into two groups based on their genome size. Phages from the *Efquatrovirus* genus belong to group one with the phages from *Phifelvirus* genus and their genome sizes range between 21 and 43 kb and contain on average 62 ORFs. On the contrary, phages from the *Saphexavirus* genus belong to group 2 with *Andrewesvirinae* subfamily. Their genome sizes range between 55 and 86 kb and they have on average 104 ORFs [38]. The organization of *Saphexavirus* and *Efquatrovirus* genomes and tail modules are typical for known *Enterococcus*-specific phages with siphovirus morphology. This same genome organization was identified also in the phages characterized in this work and their genome sizes aligned with previously classified phages from the same genera [38]. *Efquatrovirus* particles have an isometric capsid and a non-contractile tail, similar to the morphologies of fHoEfa03 and fHoEfa04 [39, 40]. The *Saphexavirus* particles contain an elongated capsid and a non-contractile tail that was seen in the micrographs taken of fHoEfa01 and fHoEfa06 [40]. The fHoEfm07 was different from earlier published phages, although its genome organization and morphology resembled the typical characteristics of siphoviruses. fHoEfm07 proteomics analysis identified structural proteins, proteins associated with DNA replication, and one hypothetical protein. These results support the functional annotation of many structural ORFs in fHoEfm07 genome. The non-structural proteins identified during the analysis participate in key-functions in initiating phage genome replication (ERF, DNA binding domain protein [41, 42]. However, we cannot exclude the possibility that these proteins are purification by-products and not included in phage capsids. As expected, the structural proteins have formed majority of identified proteins in previous studies characterizing phages using LC-MS/MS [43–46].

The phages did not carry known characteristics associated with toxins or a lysogenic life cycle. fHoEfa01, fHoEfa06 and fHoEfm07 did not carry any known AMR-related genes either, and thus, we concluded that they met the primary requisites for therapeutic use. However, the fHoEfa03 and fHoEfa04 carried a metal-dependent hydrolase potentially linked to β-lactamase resistance. The role of such hydrolases in antibiotic resistance remains unclear, and this gene was not identified by the CARD tool during screening [13]. Many *Enterococcus* phages with siphovirus morphologies that belong to the Orthocluster I, including phages belonging to the *Efquatrovirus* genus, possess this gene, and this is true as well for phages LY0322 (GenBank: MH193369.1) and PMBT2 (GenBank: MG708276.1) that are close relatives of fHoEfa03 and fHoEfa04, respectively [13]. These phages may not be ideal for therapeutic application until the implications of metal-dependent hydrolases in phage genomes are deemed harmless.

Previous studies with phages in the *Saphexavirus* genus demonstrate their potential for therapeutic applications, with a 60–100% recovery rate in test animals with a single dose of a monophage preparation [47]. Phages in the *Efquatrovirus* genus, however, have been less frequently involved in published in vivo and case studies. In one case study, a phage cocktail containing three phages that belonged to genus *Efquatrovirus*, was administered to a patient with a prosthetic UTI, but no clinical improvement was observed despite successful in vitro infection efficacy with the phage cocktail, antibiotics, and their combination [48].

The MLST typing showed that the four isolation strains belonged to three different STs. The #6467 and #7186 belonged to ST16, which includes strains found commonly from hospitals and communities and some isolates have been isolated even from livestock [49, 50]. The *E. faecalis* strain #6933 belonged to ST 81, which has also been associated with the hospital environment. The results align with the origin of the *E. faecalis* strains used for phage isolation in this work. The *E. faecium* strain #5864 belonged to ST 2026, and previous studies lack information about this ST. In addition, all *E. faecalis* strains, regardless their sequence type, carried the same incomplete prophage region. In previous studies the same intact prophage region has been found, for example, in *E. faecalis* strains isolated from poultry in Canada and an *E. faecium* strain isolated from Korean soy-meju suggesting that it is a wide-spread prophage among Enterococci [51, 52].

The host ranges of the isolated phages were relatively narrow and were not determined by the genera they were classified in. All but one of the strains used for host range screening originated from clinical environment. None of the isolated phages infected the *E. faecalis* strain isolated from pig and it seems that the phages infect clinical isolates although a more comprehensive set of strains would be needed to confirm this observation. The Enterococcus strains were obtained over a nine-year period from several hospital units. This variety in the strains may explain the narrow host specifities of the phages. Moreover, we also hypothesized that differences in the host ranges could be explained by the differences in C-terminal end of phage tail endopeptidases. The conserved domains from the endopeptidases differed between the phage genera, so that fHoEfa01 and fHoEfa06 had several conserved domains while fHoEfa03, fHoEfa04 and fHoEfm07 contained only the phage endopeptidase domain: Pfam: PF06605. Pfam: PF06605 domain is present in all endopeptidases [38]. These enzymes cleave peptide stem or cross-bridge peptide bonds of the peptidoglycan, facilitating phage genome entry. Potentially, differences in the endopeptidase genes correlate with differences in the host specificity of *Enterococcus* phages but the correlation remains hypothetical before further studies [38]. However, previous studies have demonstrated that the number and type of RBPs of otherwise similar phages result in different host specificities [53]. From the five phages isolated in this work, only fHoEfm07 showed cross-infectivity between the two *Enterococcus* species, even though its host specificity was narrow. While *Enterococcus*-phages with siphovirus morphotypes have been sometimes shown to infect across species borders, the phages with myovirus morphotypes cross-infect *E. faecalis* and *E. faecium* strains more systematically [39, 53, 54].

When combined into cocktails, the *E. faecalis*-specific phages showed diverse efficacies that depended more on the target strain than on differences in the number of phages in the cocktail or on combining phages belonging to different genera. Our data shows that different phage genera or increasing the number of phages did not always improve the cocktail efficacy, even though the phage RBPs differed between the phages. Thus, a more in-depth examination of the phage-phage interactions should be carried out to understand these interactions better. The phage-cocktail assay showed high standard deviation values during the growth of phage-resistant bacterial mutants. Such variability indicated that a specific treatment may lead to highly diverse responses in the same bacteria under the same conditions. Previous studies have identified factors leading to antagonistic phage-phage interactions, including the use of phages with similar tail fibers or phages belonging to the same family. The number of phages has been found to affect the cocktail efficacy, but its effect was not as important determinant as the phage family [4, 11]. Phage-phage interactions may not be this straightforward, as only a limited number of cocktail designs have been studied. For example, *Enterococcus* phages EFLK1 and EFDG1, from the *Spounavirinae* subfamily, infected the wildtype strain and phage-resistant mutants better as a cocktail than single phages. Although from the same subfamily, the EFLK1 and EFDG1 had different infection efficacies during different growth phases of their host, enabling the synergistic interaction [55].

Combining phages into cocktails is one strategy to overcome phage resistance, alongside using phage-antibiotic combination treatment. We studied the phage-antibiotic combinations only with the fHo-Efm07 phage due to the susceptibility of the *E. faecalis* strains against the tested antibiotics. Combining fHo-Efm07 with daptomycin or vancomycin can be an effective treatment strategy with the optimal antibiotic concentration determined before treatment. This study illustrated the success of a phage-antibiotic combination treatment, even against strains that were initially resistant to the specific antibiotic. However, the antibiotic concentrations and growth conditions used in our study differed from the European Committee on Antimicrobial Susceptibility Testing (EUCAST) guidelines. Therefore, our data is not directly comparable with the EUCAST breakpoints. Strain #5900 had earlier been confirmed as a VRE isolate. Previous studies have shown synergistic interactions between *Enterococcus* phages and antibiotics, for example with β-lactams and daptomycin [56–58]. Interestingly, Coyne et al. (2024) showed results that deviated partially from ours; phage-daptomycin synergy against *E. faecium* improved in all test strains when increasing both phage and daptomycin concentration [59]. Moreover, phage- and daptomycin-resistance may not be as concerning during combination treatment since a connection between the development of phage-resistance followed by daptomycin re-sensitization was discovered [60, 61]. Vancomycin-phage synergy has been observed in *E. faecalis* and *S. aureus*-specific phages, but it is less studied in *E. faecium*, and the mechanism of synergy is still unknown [3, 62, 63]. It has been suggested that sublethal concentration of certain antibiotics, including vancomycin, induce cellular processes that can result in increased phage production and a synergistic effect [64]. Other possibilities are that phage-antibiotic combinations cause a lot of pressure on the bacterial cell wall resulting in synergy and that phage-encoded endolysins may contribute to weakening the bacterial cell wall. However, sometimes a single antibiotic and a single phage does not fully prevent bacterial growth [65]. With fHoEfm07, the regrowth might be due to emerging resistance over time, regardless of the initial synergistic interactions. Stellfox et al. (2024) observed synergy during the treatment of recurrent *E. faecium* bacteremia between siphoviruses and daptomycin and vancomycin. The treatment resulted in clinical improvement and reduced hospitalization periods, but did not completely eradicate the pathogen, regardless of the efficacy of antibiotics, phages, and their combination in vitro. The authors suggest that the patient’s immune system affected the treatment success and reported a 10-fold decrease in phage activity in the presence of commercial pooled human serum [66].

While Stellfox et al. observed a 10-fold decrease in phage efficacy due to the unspecific effect of serum, our study showed that the effect of human serum depended on the bacterial strain and serum concentration, and whether the serum was heat-inactivated or not. The presence of NHS and HIS often inhibited the *E. faecalis* phage infections, apart from fHoEfa04, whose infection improved in all serum test conditions. fHoEfa03 and fHoEfa06 infection efficacies were not affected by the presence of 30% serum, even though the lower concentrations decreased their infectivity. fHoEfm07 infection was either improved or not affected by the tested serum concentrations. Mutations in for example the enterococcal polysaccharide antigen (*epa*) genes are linked with phage resistance and reduced virulence of Enterococcus cells [67, 68]. Thus, it is likely that phages could have synergistic interactions with the immune system. Even though we did not determine the mechanism of phage resistance in this study, we hypothesize that enhanced phage killing efficacy in the presence of human serum could result from such fitness tradeoffs. Previous studies on the synergy between phages and human serum mainly focus on gram-negative bacteria or *S. aureus*. Phage and human serum combination tend to have a synergistic effect against gram-negative bacteria, in contrast to their effect against *S. aureus*: The fibrinogen in human serum causes *S. aureus* cells to clump, resulting in a biofilm-like structure that inhibits phage infection. Traditionally, gram-positive bacteria were thought to be unaffected by the presence of serum, but recent evidence suggests that these interactions are strain specific [66, 69–71]. Our results support the latter theory, as the growth of the different *Enterococcus* strains varied in the tested NHS and HIS concentrations. Furthermore, previous phage neutralization studies show that exposure to phages may result in development of neutralizing antibodies, however, the susceptibility to immune-mediated clearance has been hypothesized to be phage specific [72, 73]. In a murine model, the authors showed that phages with myovirus morphology were more susceptible for immune-mediated clearance than phages with siphovirus morphology [73]. Another phage therapy case study showed that no neutralizing antibodies were detected after 20 days of iv treatment with a two-phage cocktail [6]. A study from 2011 demonstrated that phage ΦEF24C and a mutant phage ΦEF24C-P2 remained infectious against their host after incubation in human serum, collected from a healthy male volunteer, after 24 h incubation [74].

Biofilm formation makes enterococci a cause for chronic and difficult-to-treat infections. Thus, we investigated the in vitro efficacy of the phages against bacterial biofilms. fHoEfa03 was the only phage that caused statistically significant reduction in the biofilm masses after 3 h and 24 h. fHoEfa03 and fHoEfm07 caused statistically significant reduction in planktonic cell counts after 3 h treatment. We observed some reduction in biofilm masses and planktonic cell counts with other phages as well, however due to the high standard deviation values, these reductions were not statistically significant. Endolysins and endopeptidases were identified from all phage genomes but differed between the phages, which could be one explanation of the variation observed between the studied phages. The endopeptidases degrade peptide bonds and facilitate phage genome injection inside host cell while the endolysins facilitate the host lysis during the release of new virus particles [38, 75]. Both endopeptidases and endolysins are also associated with biofilm degradation and the differences in these proteins could explain biofilm assay results. Endolysins as anti-biofilm agents are a target of extensive research with promising results, but there is less evidence on the anti-biofilm activity of endopeptidases. The existing studies, however, show that both enzymes eradicate biofilms and reduce bacterial colonization [76–78].

Previous biofilm studies performed using microplate assays and *E. faecalis*-specific siphoviruses report biofilm mass reduction with 3 h phage incubation times but show variation after 24 h incubation. Similarly to phage fHoEfa03, another *Efquatrovirus* named Max degraded biofilms only at 3 h but not at 24 h time points. In another study, the biofilms treated with an *E. faecalis* siphovirus WH1, a close relative of our phages from the *Saphexavirus* genus, was effective even after 24 h incubation [53, 79, 80]. Moreover, we show a limited efficacy of fHoEfm07 against *E. faecium* biofilms, but other studies targeting *E. faecium* demonstrate the advances of phages for biofilm degradation, especially in combination with antibiotics [15, 81]. The lack of standardized protocols for biofilm assays complicates comparisons between existing studies. Therefore, there is a serious need for developing reproducible and standardized protocols.

## Conclusion

Publications on *Enterococcus*-phages and their suitability for therapeutic applications cover a limited number of phages and experimental designs. This study demonstrates the potential of phage therapy in addressing *Enterococcus* infections. Especially fHoEfa01, fHoEfa06 and fHoEfm07 show promise for therapeutic applications due to their lytic nature and absence of harmful genes. However, the presence of metal-dependent hydrolases in the genomes of fHoEfa03 and fHoEfa04 necessitates further investigation to ensure safety. On the other hand, their efficacy against bacterial biofilms would be beneficial in, for example, health care and water infrastructure applications. Our study highlights the complexity of phage-phage and phage-host interactions, emphasizing the need for more in vitro and in vivo experimental data before we can apply computational and automated approaches to improve phage characterization and phage therapy.

## Supplementary Information

Supplementary Material 1

## Data Availability

This publication and its supplementary information files include all data presented in this study. The genomic sequences of the phages isolated in this study have been submitted to the NCBI GenBank, https://www.ncbi.nlm.nih.gov/genbank/, under the following accession numbers: fHoEfa01: PV296017, fHoEfa03: PV296018, fHoEfa04: PV296019, fHoEfa06: PV296020 and fHoEfa07: PV296021. The raw sequencing data of the isolation hosts were submitted to the Sequence Reads Archive (SRA), https://www.ncbi.nlm.nih.gov/sra, BioProject ID PRJNA1355155, BioSamples SAMN52968961 for #6467, SAMN52968962 for #6933, SAMN52968963 for #7186 and SAMN52968964 for #5864. Further inquiries can be directed to the corresponding author.

## References

[1] Gao W, Howden BP, Stinear TP. Evolution of virulence in *Enterococcus faecium*, a hospital-adapted opportunistic pathogen. Curr Opin Microbiol. 2018;41:76–82. 29227922 10.1016/j.mib.2017.11.030

[2] Arias CA, Murray BE. The rise of the *Enterococcus*: beyond vancomycin resistance. Nat Rev Microbiol. 2012;10(4):266–78. 22421879 10.1038/nrmicro2761 PMC3621121

[3] Shlezinger M, Coppenhagen-Glazer S, Gelman D, Beyth N, Hazan R. Eradication of Vancomycin-Resistant Enterococci by Combining Phage and Vancomycin. Viruses. 2019. 10.3390/v11100954. 10.3390/v11100954 PMC6833023 31623253

[4] Wandro S, Ghatbale P, Attai H, Hendrickson C, Samillano C, Suh J, et al. Phage Cocktails Constrain Growth Enterococcus mSystems. 2022;7(4):e0001922. 35762793 10.1128/msystems.00019-22 PMC9426582

[5] Letkiewicz S, Miedzybrodzki R, Fortuna W, Weber-Dabrowska B, Górski A. Eradication of *Enterococcus faecalis* by phage therapy in chronic bacterial prostatitis–case report. Folia Microbiol (Praha). 2009;54(5):457–61. 19937220 10.1007/s12223-009-0064-z

[6] Paul K, Merabishvili M, Hazan R, Christner M, Herden U, Gelman D, et al. Bacteriophage rescue therapy of a vancomycin-resistant *Enterococcus faecium* infection in a one-year-old child following a third liver transplantation. Viruses. 2021;13(9):1785. 34578366 10.3390/v13091785 PMC8472888

[7] Morrisette T, Lev KL, Kebriaei R, Abdul-Mutakabbir JC, Stamper KC, Morales S, et al. Bacteriophage-Antibiotic Combinations for Enterococcus faecium with Varying Bacteriophage and Daptomycin Susceptibilities. Antimicrob Agents Chemother. 2020. 10.1128/AAC.00993-20. 10.1128/AAC.00993-20 PMC7449186 32571816

[8] Moryl M, Różalski A, de Figueiredo JAP, Palatyńska-Ulatowska A. How do phages disrupt the structure of *Enterococcus faecalis* biofilm? Int J Mol Sci. 2023;24(24):17260. 38139094 10.3390/ijms242417260 PMC10744153

[9] Morrisette T, Lev KL, Canfield GS, Duerkop BA, Kebriaei R, Stamper KC, et al. Evaluation of bacteriophage cocktails alone and in combination with daptomycin against daptomycin-nonsusceptible *Enterococcus faecium*. Antimicrob Agents Chemother. 2022;66(1):e0162321. 34723631 10.1128/AAC.01623-21 PMC8765229

[10] Nir-Paz R, Gelman D, Khouri A, Sisson BM, Fackler J, Alkalay-Oren S, et al. Successful treatment of antibiotic-resistant, poly-microbial bone infection with bacteriophages and antibiotics combination. Clin Infect Dis. 2019;69(11):2015–8. 30869755 10.1093/cid/ciz222

[11] Kim MK, Chen Q, Echterhof A, Pennetzdorfer N, McBride RC, Banaei N, et al. A blueprint for broadly effective bacteriophage-antibiotic cocktails against bacterial infections. Nat Commun. 2024;15(1):9987. 39609398 10.1038/s41467-024-53994-9 PMC11604943

[12] Oechslin F. Resistance Development to Bacteriophages Occurring during Bacteriophage Therapy. Viruses. 2018. 10.3390/v10070351. 10.3390/v10070351 PMC6070868 29966329

[13] Bolocan AS, Upadrasta A, Bettio PHA, Clooney AG, Draper LA, Ross RP, et al. Evaluation of Phage Therapy in the Context of Enterococcus faecalis and Its Associated Diseases. Viruses. 2019. 10.3390/v11040366. 10.3390/v11040366 PMC6521178 31010053

[14] Haack S, Duris J. Dynamics of fecal indicator bacteria, bacterial pathogen genes, and organic wastewater contaminants in the Little Calumet River: portage burns waterway, Indiana. J Great Lakes Res. 2013;39(2):317–26.

[15] Lev K, Kunz Coyne AJ, Kebriaei R, Morrisette T, Stamper K, Holger DJ, et al. Evaluation of Bacteriophage-Antibiotic Combination Therapy for Biofilm-Embedded MDR Enterococcus faecium. Antibiotics. 2022. 10.3390/antibiotics11030392. 10.3390/antibiotics11030392 PMC8944492 35326855

[16] Mangalea Mihnea R, Duerkop Breck A. Fitness Trade-Offs Resulting from Bacteriophage Resistance Potentiate Synergistic Antibacterial Strategies. Infect Immun. 2020;88(7). 10.1128/IAI.00926-19 PMC7309606 32094257

[17] Naghizadeh M, Karimi Torshizi MA, Rahimi S, Engberg RM, Sørensen Dalgaard T. Effect of serum anti-phage activity on colibacillosis control by repeated phage therapy in broilers. Vet Microbiol. 2019;234:61–71. 31213273 10.1016/j.vetmic.2019.05.018

[18] Ribes-Martínez L, Muñoz-Egea M-C, Yuste J, Esteban J, García-Quintanilla M. Bacteriophage therapy as a promising alternative for antibiotic-resistant *Enterococcus faecium*: advances and challenges. Antibiotics (Basel). 2024;13(12):1120. 39766510 10.3390/antibiotics13121120 PMC11672805

[19] Sambrook J, Russel DW. Molecular Cloning: A Laboratory Manual. 3rd ed. New York: Cold Spring Harbor Laboratory Press; 2001.

[20] Tuomala H, Verkola M, Meller A, Van der Auwera J, Patpatia S, Järvinen A, et al. Phage Treatment Trial to Eradicate LA-MRSA from Healthy Carrier Pigs. Viruses. 2021. 10.3390/v13101888. 10.3390/v13101888 PMC8539482 34696318

[21] Tritt A, Eisen JA, Facciotti MT, Darling AE. An integrated pipeline for de novo assembly of microbial genomes. PLoS One. 2012;7(9):e42304. 23028432 10.1371/journal.pone.0042304 PMC3441570

[22] Garneau JR, Depardieu F, Fortier L-C, Bikard D, Monot M. PhageTerm: a tool for fast and accurate determination of phage termini and packaging mechanism using next-generation sequencing data. Sci Rep. 2017;7(1):8292. 28811656 10.1038/s41598-017-07910-5 PMC5557969

[23] Arndt D, Grant JR, Marcu A, Sajed T, Pon A, Liang Y, et al. PHASTER: a better, faster version of the PHAST phage search tool. Nucleic Acids Res. 2016;44(W1):W16-21. 27141966 10.1093/nar/gkw387 PMC4987931

[24] Alcock BP, Huynh W, Chalil R, Smith KW, Raphenya AR, Wlodarski MA, et al. CARD 2023: expanded curation, support for machine learning, and resistome prediction at the Comprehensive Antibiotic Resistance Database. Nucleic Acids Res. 2023;51(D1):D690–9. 36263822 10.1093/nar/gkac920 PMC9825576

[25] Aziz RK, Bartels D, Best AA, DeJongh M, Disz T, Edwards RA, et al. The RAST server: rapid annotations using subsystems technology. BMC Genomics. 2008;9(1):75. 18261238 10.1186/1471-2164-9-75 PMC2265698

[26] Nishimura Y, Yoshida T, Kuronishi M, Uehara H, Ogata H, Goto S. ViPTree: the viral proteomic tree server. Bioinformatics. 2017;33(15):2379–80. 28379287 10.1093/bioinformatics/btx157

[27] Letunic I, Bork P. Interactive tree of life (iTOL) v6: recent updates to the phylogenetic tree display and annotation tool. Nucleic Acids Res. 2024;52(W1):W78-82. 38613393 10.1093/nar/gkae268 PMC11223838

[28] Nishimura Y, Yamada K, Okazaki Y, Ogata H. DiGAlign: Versatile and Interactive Visualization of Sequence Alignment for Comparative Genomics. Microbes Environ. 2024. 10.1264/jsme2.ME23061. 10.1264/jsme2.ME23061 PMC10982109 38508742

[29] Katoh K, Standley DM. MAFFT multiple sequence alignment software version 7: improvements in performance and usability. Mol Biol Evol. 2013;30(4):772–80. 23329690 10.1093/molbev/mst010 PMC3603318

[30] Price MN, Dehal PS, Arkin AP. FastTree: computing large minimum evolution trees with profiles instead of a distance matrix. Mol Biol Evol. 2009;26(7):1641–50. 19377059 10.1093/molbev/msp077 PMC2693737

[31] Olson RD, Assaf R, Brettin T, Conrad N, Cucinell C, Davis James J, et al. Introducing the Bacterial and Viral Bioinformatics Resource Center (BV-BRC): a resource combining PATRIC, IRD and ViPR. Nucleic Acids Res. 2022;51(D1):D678–89. 10.1093/nar/gkac1003 PMC9825582 36350631

[32] Brettin T, Davis JJ, Disz T, Edwards RA, Gerdes S, Olsen GJ, et al. RASTtk: a modular and extensible implementation of the RAST algorithm for building custom annotation pipelines and annotating batches of genomes. Sci Rep. 2015;5(1):8365. 25666585 10.1038/srep08365 PMC4322359

[33] Overbeek R, Olson R, Pusch GD, Olsen GJ, Davis JJ, Disz T, et al. The SEED and the Rapid Annotation of microbial genomes using Subsystems Technology (RAST). Nucleic Acids Res. 2013;42(D1):D206–14. 24293654 10.1093/nar/gkt1226 PMC3965101

[34] Zhou Y, Liang Y, Lynch KH, Dennis JJ, Wishart DS. PHAST: a fast phage search tool. Nucleic Acids Res. 2011;39(suppl2):W347-52. 21672955 10.1093/nar/gkr485 PMC3125810

[35] Tesson F, Hervé A, Mordret E, Touchon M, d’Humières C, Cury J, et al. Systematic and quantitative view of the antiviral arsenal of prokaryotes. Nat Commun. 2022;13(1):2561. 35538097 10.1038/s41467-022-30269-9 PMC9090908

[36] Tisza MJ, Buck CB. A catalog of tens of thousands of viruses from human metagenomes reveals hidden associations with chronic diseases. Proc Natl Acad Sci U S A. 2021;118(23):e2023202118. 34083435 10.1073/pnas.2023202118 PMC8201803

[37] Pchelin IM, Tkachev PV, Azarov DV, Gorshkov AN, Drachko DO, Zlatogursky VV, et al. A genome of temperate *Enterococcus* bacteriophage placed in a space of pooled viral dark matter sequences. Viruses. 2023;15(1):216. 36680256 10.3390/v15010216 PMC9865981

[38] Alrafaie AM, Stafford GP. Enterococcal bacteriophage: a survey of the tail associated lysin landscape. Virus Res. 2023;327:199073. 36787848 10.1016/j.virusres.2023.199073 PMC10194240

[39] Alrafaie AM, Pyrzanowska K, Smith EM, Partridge DG, Rafferty J, Mesnage S, et al. A diverse set of *Enterococcus*-infecting phage provides insight into phage host-range determinants. Virus Res. 2024;347:199426. 38960003 10.1016/j.virusres.2024.199426 PMC11269942

[40] Di Lallo G, Falconi M, Iacovelli F, Frezza D, D’Addabbo P. Analysis of four new *Enterococcus faecalis* phages and modeling of a hyaluronidase catalytic domain from saphexavirus. Phage (New Rochelle). 2021;2(3):131–41. 36161247 10.1089/phage.2021.0003 PMC9041502

[41] Guliy OI, Evstigneeva SS. Bacteria- and Phage-Derived Proteins in Phage Infection. Frontiers in Bioscience-Landmark. 2025. 10.31083/FBL24478. 10.31083/FBL24478 40018916

[42] Poteete AR, Fenton AC. DNA-binding properties of the Erf protein of bacteriophage P22. J Mol Biol. 1983;163(2):257–75. 6302269 10.1016/0022-2836(83)90006-2

[43] Topka-Bielecka G, Bloch S, Nejman-Faleńczyk B, Grabski M, Jurczak-Kurek A, Górniak M, et al. Characterization of the Bacteriophage vB_EfaS-271 Infecting Enterococcus faecalis. Int J Mol Sci. 2020;21(17):6345. 32882938 10.3390/ijms21176345 PMC7503890

[44] Parasion S, Kwiatek M, Mizak L, Gryko R, Bartoszcze M, Kocik J. Isolation and characterization of a novel bacteriophage φ4D lytic against *Enterococcus faecalis* strains. Curr Microbiol. 2012;65(3):284–9. 22669253 10.1007/s00284-012-0158-8

[45] Khazani Asforooshani M, Elikaei A, Abed S, Shafiei M, Barzi SM, Solgi H et al. A novel Enterococcus faecium phage EF-M80: unveiling the effects of hydrogel-encapsulated phage on wound infection healing. Front Microbiol. 2024;Frontiers in Microbiology, 2024. Volume 15 - 2024. 10.3389/fmicb.2024.1416971 10.3389/fmicb.2024.1416971 PMC11239553 39006751

[46] Lavigne R, Ceyssens P-J, Robben J. Phage Proteomics: Applications of Mass Spectrometry. In: Clokie MRJ, Kropinski AM, editors. Bacteriophages: Methods and Protocols, Volume 2 Molecular and Applied Aspects. Totowa, NJ: Humana; 2009. pp. 239–51. 10.1007/978-1-60327-565-1_14 19082560

[47] Cheng M, Liang J, Zhang Y, Hu L, Gong P, Cai R, et al. The Bacteriophage EF-P29 Efficiently Protects against Lethal Vancomycin-Resistant Enterococcus faecalis and Alleviates Gut Microbiota Imbalance in a Murine Bacteremia Model. Front Microbiol. 2017;8:2017. 28536572 10.3389/fmicb.2017.00837 PMC5423268

[48] Stevens RH, Zhang H, Kajsik M, Płoski R, Rydzanicz M, Sabaka P et al. Successful use of a phage endolysin for treatment of chronic pelvic pain syndrome/chronic bacterial prostatitis. Front Med. 2023;Volume: 10–2023. 10.3389/fmed.2023.1238147 10.3389/fmed.2023.1238147 PMC10462781 37649979

[49] Nüesch-Inderbinen M, Raschle S, Stevens MJA, Schmitt K, Stephan R. Linezolid-resistant *Enterococcus faecalis* ST16 harbouring optrA on a Tn6674-like element isolated from surface water. J Glob Antimicrob Resist. 2021;25:89–92. 33705941 10.1016/j.jgar.2021.02.029

[50] Quiñones D, Kobayashi N, Nagashima S. Molecular epidemiologic analysis of *Enterococcus faecalis* isolates in Cuba by multilocus sequence typing. Microb Drug Resist. 2009;15(4):287–93. 19857135 10.1089/mdr.2009.0028 PMC3145955

[51] Kim DH, Kim SA, Jo NG, Bae JH, Nguyen MT, Jo YM, et al. Phenotypic and genomic analyses of bacteriocin-producing probiotic *Enterococcus faecium* EFEL8600 isolated from Korean soy-meju. Front Microbiol. 2023;14:1237442. 37731927 10.3389/fmicb.2023.1237442 PMC10507247

[52] Deslauriers N, Boulianne M. Genetic comparison of *Enterococcus* species isolated from osteomyelitis lesions and the barn environment of successive broiler chicken flocks. Avian Dis. 2025;68(S1):421–6. 40249581 10.1637/aviandiseases-D-24-00081

[53] Al-Zubidi M, Widziolek M, Court EK, Gains AF, Smith RE, Ansbro K, et al. Identification of Novel Bacteriophages with Therapeutic Potential That Target Enterococcus faecalis. Infect Immun. 2019. 10.1128/IAI.00512-19. 10.1128/IAI.00512-19 PMC6803325 31451618

[54] Zhang W, Mi Z, Yin X, Fan H, An X, Zhang Z, et al. Characterization of *Enterococcus faecalis* phage IME-EF1 and its endolysin. PLoS One. 2013;8(11):e80435. 24236180 10.1371/journal.pone.0080435 PMC3827423

[55] Khalifa L, Gelman D, Shlezinger M, Dessal AL, Coppenhagen-Glazer S, Beyth N, et al. Defeating Antibiotic- and Phage-Resistant Enterococcus faecalis Using a Phage Cocktail in Vitro and in a Clot Model. Front Microbiol. 2018;9:2018. 29541067 10.3389/fmicb.2018.00326 PMC5835721

[56] Moryl M, Szychowska P, Dziąg J, Różalski A, Torzewska A. The combination of phage therapy and β-lactam antibiotics for the effective treatment of *Enterococcus faecalis* infections. Int J Mol Sci. 2025;26(1):11. 10.3390/ijms26010011 PMC11719584 39795870

[57] Canfield GS, Chatterjee A, Espinosa J, Mangalea MR, Sheriff EK, Keidan M, et al. Lytic bacteriophages facilitate antibiotic sensitization of Enterococcus faecium. Antimicrob Agents Chemother. 2021. 10.1128/AAC.00143-21. 10.1128/AAC.00143-21 PMC8092871 33649110

[58] Kunz Coyne AJ, Stamper K, El Ghali A, Kebriaei R, Biswas B, Wilson M, et al. Phage-antibiotic cocktail rescues daptomycin and phage susceptibility against daptomycin-nonsusceptible *Enterococcus faecium* in a simulated endocardial vegetation ex vivo model. Microbiol Spectr. 2023;11(4):e0034023. 37338375 10.1128/spectrum.00340-23 PMC10433949

[59] Kunz Coyne AJ, Eshaya M, Bleick C, Vader S, Biswas B, Wilson M, et al. Exploring synergistic and antagonistic interactions in phage-antibiotic combinations against ESKAPE pathogens. Microbiol Spectr. 2024;12(10):e0042724. 39082827 10.1128/spectrum.00427-24 PMC11468199

[60] Fujiki J, Nakamura K, Nakamura T, Iwano H. Fitness Trade-Offs between Phage and Antibiotic Sensitivity in Phage-Resistant Variants: Molecular Action and Insights into Clinical Applications for Phage Therapy. Int J Mol Sci. 2023. 10.3390/ijms242115628. 10.3390/ijms242115628 PMC10650657 37958612

[61] Ho K, Huo W, Pas S, Dao R, Palmer KL. Loss-of-Function Mutations in < i>epaRConfer Resistance to ϕNPV1 Infection in Enterococcus faecalis OG1RF. Antimicrobial Agents and Chemotherapy. 2018;62(10):10.1128/aac.00758 – 18. 10.1128/AAC.00758-18 PMC6153818 30104266

[62] Taha M, Arnaud T, Lightly TJ, Peters D, Wang L, Chen W et al. Combining bacteriophage and vancomycin is efficacious against MRSA biofilm-like aggregates formed in synovial fluid. Front Med. 2023; Volume : 10–2023. 10.3389/fmed.2023.1134912 10.3389/fmed.2023.1134912 PMC10289194 37359001

[63] Sahu M, Vishwakarma RK, Karn SL, Nath G. Synergistic efficacy of phages along with vancomycin for eradication of vancomycin-resistant *Enterococcus faecalis* biofilms. World J Virol. 2025;14(2):95826. 40575641 10.5501/wjv.v14.i2.95826 PMC12188910

[64] Shlezinger M, Coppenhagen-Glazer S, Gelman D, Beyth N, Hazan R. Eradication of vancomycin-resistant enterococci by combining phage and vancomycin. Viruses. 2019;11(10):954. 31623253 10.3390/v11100954 PMC6833023

[65] Loganathan A, Bozdogan B, Manohar P, Nachimuthu R. Phage-antibiotic combinations in various treatment modalities to manage MRSA infections. Front Pharmacol. 2024;Volume : 15–2024. 10.3389/fphar.2024.1356179 10.3389/fphar.2024.1356179 PMC11041375 38659581

[66] Stellfox ME, Fernandes C, Shields RK, Haidar G, Hughes Kramer K, Dembinski E, et al. Bacteriophage and antibiotic combination therapy for recurrent *Enterococcus faecium* bacteremia. MBio. 2024;15(3):e0339623. 38353560 10.1128/mbio.03396-23 PMC10936196

[67] Canfield Gregory S, Chatterjee A, Espinosa J, Mangalea Mihnea R, Sheriff Emma K, Keidan M, et al. Lytic Bacteriophages Facilitate Antibiotic Sensitization of Enterococcus faecium. Antimicrob Agents Chemother. 2021;65(5). 10.1128/AAC.00143-21 PMC8092871 33649110

[68] Rigottier-Gois L, Madec C, Navickas A, Matos RC, Akary-Lepage E, Mistou MY, et al. The surface rhamnopolysaccharide epa of Enterococcus faecalis is a key determinant of intestinal colonization. J Infect Dis. 2015;211(1):62–71. 25035517 10.1093/infdis/jiu402

[69] Tickle ARH, Ledger EVK, Edwards AM. Human serum induces daptomycin tolerance in Enterococcus faecalis and viridans group streptococci. Microbiol (Reading). 2022;168(12). 10.1099/mic.0.001282 36748501

[70] Mutti M, Moreno DS, Restrepo-Córdoba M, Visram Z, Resch G, Corsini L. Phage activity against *Staphylococcus aureus* is impaired in plasma and synovial fluid. Sci Rep. 2023;13(1):18204. 37875544 10.1038/s41598-023-45405-8 PMC10598271

[71] Nallapareddy SR, Murray BE. Role played by serum, a biological cue, in the adherence of *Enterococcus faecalis* to extracellular matrix proteins, collagen, fibrinogen, and fibronectin. J Infect Dis. 2008;197(12):1728–36. 18462135 10.1086/588143 PMC2735109

[72] Łusiak-Szelachowska M, Żaczek M, Weber-Dąbrowska B, Międzybrodzki R, Kłak M, Fortuna W, et al. Phage neutralization by sera of patients receiving phage therapy. Viral Immunol. 2014;27(6):295–304. 24893003 10.1089/vim.2013.0128 PMC4076984

[73] Berkson JD, Wate CE, Allen GB, Schubert AM, Dunbar KE, Coryell MP, et al. Phage-specific immunity impairs efficacy of bacteriophage targeting Vancomycin Resistant Enterococcus in a murine model. Nature Communications. 2024;15(1):2993. 10.1038/s41467-024-47192-w PMC10998888 38582763

[74] Uchiyama J, Takemura I, Satoh M, Kato S-i, Ujihara T, Akechi K, et al. Improved adsorption of an *Enterococcus faecalis* bacteriophage ΦEF24C with a spontaneous point mutation. PLoS ONE. 2011;6(10):e26648. 22046321 10.1371/journal.pone.0026648 PMC3201976

[75] Xiang Y, Wang S, Huang H, Li X, Wei Y, Li H, et al. A novel endolysin from an *Enterococcus faecalis* phage and application. Microb Pathog. 2024;192:106689. 38750777 10.1016/j.micpath.2024.106689

[76] Fenton M, Keary R, McAuliffe O, Ross RP, O’Mahony J, Coffey A. Bacteriophage-derived peptidase CHAP(K) eliminates and prevents staphylococcal biofilms. Int J Microbiol. 2013;2013:625341. 23431312 10.1155/2013/625341 PMC3574654

[77] Oh HK, Hwang YJ, Hong HW, Myung H. Comparison of Enterococcus faecalis Biofilm Removal Efficiency among Bacteriophage PBEF129, Its Endolysin, and Cefotaxime. Viruses. 2021. 10.3390/v13030426. 10.3390/v13030426 PMC7999683 33800040

[78] Wang J, Liang S, Lu X, Xu Q, Zhu Y, Yu S et al. Bacteriophage endolysin Ply113 as a potent antibacterial agent against polymicrobial biofilms formed by enterococci and Staphylococcus aureus. Front Microbiol. 2023;Volume: 14–2023. 10.3389/fmicb.2023.1304932 10.3389/fmicb.2023.1304932 PMC10751913 38152375

[79] Melo LDR, Ferreira R, Costa AR, Oliveira H, Azeredo J. Efficacy and safety assessment of two enterococci phages in an in vitro biofilm wound model. Sci Rep. 2019;9(1):6643. 31040333 10.1038/s41598-019-43115-8 PMC6491613

[80] Jin X, Sun X, Wang Z, Dou J, Lin Z, Lu Q, et al. Virulent Phage vB_EfaS_WH1 Removes Enterococcus faecalis Biofilm and Inhibits Its Growth on the Surface of Chicken Meat. Viruses. 2023. 10.3390/v15051208. 10.3390/v15051208 PMC10221825 37243294

[81] Goodarzi F, Hallajzadeh M, Sholeh M, Talebi M, Mahabadi VP, Amirmozafari N. Biological characteristics and anti-biofilm activity of a lytic phage against vancomycin-resistant *Enterococcus faecium*. Iran J Microbiol. 2021;13(5):691–702. 34900167 10.18502/ijm.v13i5.7436 PMC8629820

