# Supplementary material for: Antibacterial potential of five phages in controlling Enterococcus faecalis and Enterococcus faecium
Source: Virol J. 2026 Jun 2;23:143. doi: 10.1186/s12985-026-03147-9 (PMC13227665; doi:10.1186/s12985-026-03147-9)
Supplement: Supplementary file 1 — Supplementary Material 1 [file 12985_2026_3147_MOESM1_ESM.docx]

**Antibacterial Potential of Five Phages in Controlling *Enterococcus faecalis* and *Enterococcus faecium***

Henni Tuomala, Tiina Nylund, Markus Mustonen, Annika Flod, and Saija Kiljunen

**Supplementary Table 1.** *E. faecalis* and *E.faecium* strains used in this study and their susceptibility to phages fHoEfa01, fHoEfa03, fHoEfa04, fHoEfa06 and fHoEfm07.

| Species | Strain | Origin | fHoEfa01 | fHoEfa03 | fHoEfa04 | fHoEfa06 | fHoEfm07 |
| --- | --- | --- | --- | --- | --- | --- | --- |
| E. faecalis | #5498 | Clinical | + | - | - | - | - |
| E. faecalis | #5713 | Clinical | - | - | + | + | - |
| E. faecalis | #5714 | Clinical | - | - | + | + | - |
| E. faecalis | #5715 | Clinical | - | - | + | + | - |
| E. faecalis | #5716 | Clinical | - | - | + | + | - |
| E. faecalis | #5717 | Clinical | - | - | + | + | - |
| E. faecalis | #5718 | Clinical | - | - | + | + | - |
| E. faecalis | #5719 | Clinical | - | - | + | + | - |
| E. faecalis | #5720 | Clinical | - | - | + | + | - |
| E. faecalis | #5721 | Clinical | - | - | + | + | - |
| E. faecalis | #5722 | Clinical | +/- | +/- | + | + | - |
| E. faecalis | #6259 | Pig | -^*^ | -^*^ | -^*^ | - | -^*^ |
| E. faecalis | #6315 | Clinical | - | - | - | - | - |
| E. faecalis | #6467 | Clinical | +^1^ | +^1^ | - | + | - |
| E. faecalis | #6552 | Clinical | - | - | - | - | - |
| E. faecalis | #6568 | Clinical | -^*^ | -^*^ | -^*^ | - | -^*^ |
| E. faecalis | #6569 | Clinical | + | - | - | - | - |
| E. faecalis | #6570 | Clinical | - | - | - | - | +/- |
| E. faecalis | #6571 | Clinical | + | - | - | - | - |
| E. faecalis | #6908 | Clinical | - | - | - | - | - |
| E. faecalis | #6933 | Clinical | -^*^ | -^*^ | +^1^ | - | -^*^ |
| E. faecalis | #6934 | Clinical | - | - | + | +/- | - |
| E. faecalis | #7007 | Clinical | -^*^ | -^*^ | -^*^ | - | -^*^ |
| E. faecalis | #7107 | Clinical | - | - | - | - | - |
| E. faecalis | #7186 | Clinical | + | + | - | +^1^ | - |
| E. faecium | #5543 | Clinical | - | - | - | - | - |
| E. faecium | #5545 | Clinical | - | - | - | - | - |
| E. faecium | #5552 | Clinical | -^*^ | -^*^ | -^*^ | - | -^*^ |
| E. faecium | #5792 | Clinical | -^*^ | -^*^ | -^*^ | - | -^*^ |
| E. faecium | #5864 | Clinical | -^*^ | -^*^ | -^*^ | - | +^1^ |
| E. faecium | #5866 | Clinical | -^*^ | -^*^ | -^*^ | - | -^*^ |
| E. faecium | #5867 | Clinical | - | - | - | - | - |
| E. faecium | #5868 | Clinical | - | - | - | - | - |
| E. faecium | #5877 | Clinical | - | - | - | - | - |
| E. faecium | #5878 | Clinical | -^*^ | -^*^ | -^*^ | - | -^*^ |
| E. faecium | #5879 | Clinical | - | - | - | - | - |
| E. faecium | #5881 | Clinical | - | - | - | - | - |
| E. faecium | #5885 | Clinical | -^*^ | -^*^ | -^*^ | -^*^ | -^*^ |
| E. faecium | #5886 | Clinical | - | - | - | - | - |
| E. faecium | #5887 | Clinical | - | - | - | - | - |
| E. faecium | #5888 | Clinical | - | - | - | - | - |
| E. faecium | #5889 | Clinical | - | - | - | - | - |
| E. faecium | #5893 | Clinical | - | - | - | - | - |
| E. faecium | #5895 | Clinical | - | - | - | - | - |
| E. faecium | #5896 | Clinical | - | - | -^*^ | - | -^*^ |
| E. faecium | #5897 | Clinical | - | - | -^*^ | - | -^*^ |
| E. faecium | #5898 | Clinical | - | - | -^*^ | - | -^*^ |
| E. faecium | #5899 | Clinical | - | - | -^*^ | - | -^*^ |
| E. faecium | #5890 | Clinical | - | - | - | - | - |
| E. faecium | #5891 | Clinical | - | - | - | - | - |
| E. faecium | #5900 | Clinical | - | - | -^*^ | - | + |

+ Clear infection

+/- Intermediate infection

- Resistant

^1^ Isolation host

*Tested with the double-layer spot assay method

**Supplementary Figure S1**


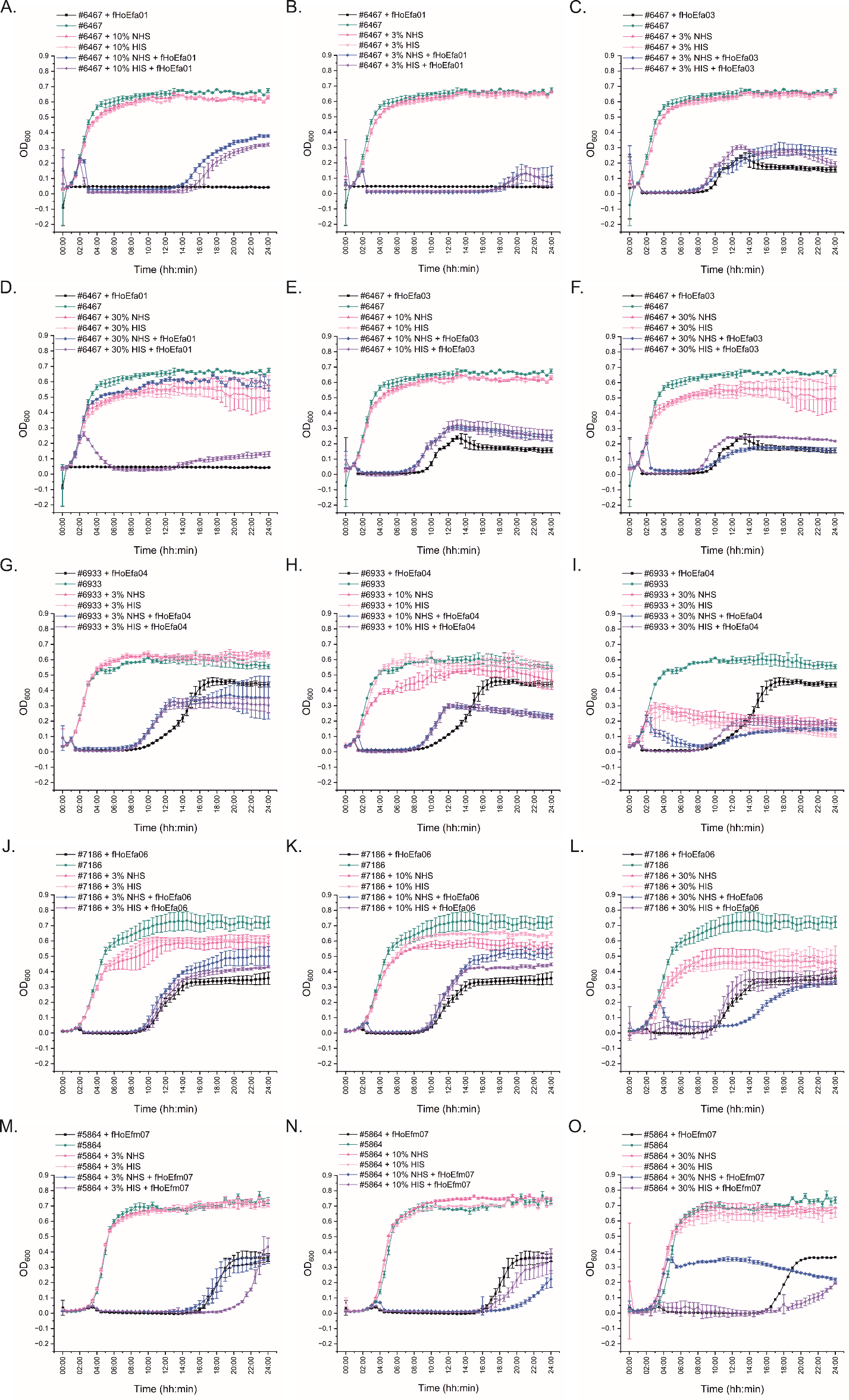


**Supplementary Figure S1 Phage-human serum interaction assay.** The phage human serum interactions were studied in 3%, 10% and 30% commercial normal human serum (NHS) and heat-inactivated human serum (HIS) using liquid assay. The bacterial growth in the presence of serum and/or phage was compared to bacterial growth alone and BHI broth was used as a negative control and the growth was measured at OD_600_ for 24h. The samples were prepared as triplicates that were used to calculate mean values and standard deviation. A-C) fHoEfa01 in the presence of NHS and HIS. D-F) fHoEfa03 in the presence of NHS and HIS. G-I) fHoEfa04 in the presence of NHS and HIS. J-L) fHoEfa06 in the presence of NHS and HIS. M-O) fHoEfm07 in the presence of NHS and HIS.

**Supplementary Table S2 Virulence genes detected from the Enterococcus strains.** Rapid Annotation Using the Subsystem Technology (RAST) online tool was used to identify virulence genes from the *Enterococcus* host strains**.**

| **Strain** | **Invasion and intracellular resistance** | **Number of genes associated with the system** |
| --- | --- | --- |
| *E. faecalis* #6467 | Mycobacterium virulence operon involved in DNA transcription | 2 |
|  | Mycobacterium virulence operon involved in protein synthesis (SSU ribosomal proteins) | 4 |
|  | Mycobacterium virulence operon involved in protein synthesis (LSU ribosomal proteins) | 3 |
| *E. faecalis* #6933 | Mycobacterium virulence operon involved in DNA transcription | 2 |
|  | Mycobacterium virulence operon involved in protein synthesis (SSU ribosomal proteins) | 4 |
|  | Mycobacterium virulence operon involved in protein synthesis (LSU ribosomal proteins) | 3 |
| *E. faecalis* #7186 | Mycobacterium virulence operon involved in DNA transcription | 2 |
|  | Mycobacterium virulence operon involved in protein synthesis (SSU ribosomal proteins) | 4 |
|  | Mycobacterium virulence operon involved in protein synthesis (LSU ribosomal proteins) | 3 |
| *E. faecium* #5864 | Mycobacterium virulence operon involved in DNA transcription | 2 |
|  | Mycobacterium virulence operon involved in protein synthesis (SSU ribosomal proteins) | 5 |
|  | Mycobacterium virulence operon involved in protein synthesis (LSU ribosomal proteins) | 3 |

**Supplementary Table S3 Prophage sequences detected from the *Enterococcus* genomes.** Phage Search Tool Enhanced Release (PHASTER) web tool was used for detection of prophage regions and evaluation of the completeness of the regions. The completeness score threshold values were: Intact (score > 90), Questionable (score 70-90), Incomplete (score < 70).

| **Strain** | **Most Common Phage name** | **Region (start position)** | **Region length (kb)** | **Completeness (score)** |
| --- | --- | --- | --- | --- |
| #6467 | PHAGE_Lactob_Lj928_NC_005354(6) | Contig 1 (618,578) | 20.4 | Intact (120) |
|  | PHAGE_Entero_vB_IME197_NC_028671(7) | Contig 2 (190,869) | 14.6 | Incomplete (40) |
| #6933 | PHAGE_Entero_vB_IME197_NC_028671(7) | Contig 1 (201,107) | 14.6 | Incomplete (40) |
| #7186 | PHAGE_Entero_vB_IME197_NC_028671(7) | Contig 1 (223,436) | 14.6 | Incomplete (40) |
|  | PHAGE_Entero_phiEf11_NC_013696(6) | Contig 1 (794,040) | 20.4 | Intact (120) |
|  | PHAGE_Entero_phiFL1A_NC_013646(7) | Contig 1 (806,525) | 34.7 | Incomplete (30) |
|  | PHAGE_Entero_EFC_1_NC_025453(41) | Contig 3 (20,438) | 66.4 | Intact (150) |
|  | PHAGE_Synech_S_T4_NC_048049(2) | Contig 6 (27,357) | 9.5 | Incomplete (30) |
| #5864 | PHAGE_Lister_B054_NC_009813(2) | Contig 1 (67,004) | 19.2 | Incomplete (30) |

**Supplementary Table S4 Antimicrobial resistance (AMR) genes identified from the genomes of the *Enterococcus* strains.** Resistance Gene Identifier (RGI) tool (v6.0.3) and the Comprehensive Antibiotic Resistance Database (CARD) (v3.2.9) were used for detecting AMR genes.

| **Strain** | **RGI Criteria** | **ARO Term** | **Drug class** | **Resistance mechanism** | **% Identity of Matching Region** | **% Length of Reference Sequence** |
| --- | --- | --- | --- | --- | --- | --- |
| #6467 | Perfect | *dfrG* | diaminopyrimidine | antibiotic target replacement | 100.0 | 100.0 |
|  | Perfect | *lsaE* | lincosamide, streptogramin, pleuromutilin | antibiotic target protection | 100.00 | 100.00 |
|  | Perfect | AAC(6')- Ie-APH(2'')- Ia bifunction al protein | aminoglycoside | antibiotic inactivation | 100.00 | 100.00 |
|  | Strict | *vanT* gene in *vanG* cluster | glycopeptide | antibiotic target alteration | 34.78 | 52.11 |
|  | Strict | *tet*(M) | tetracycline | antibiotic target protection | 94.05 | 100.00 |
|  | Strict | *dfrE* | diaminopyrimidine | antibiotic target replacement | 98.78 | 100.00 |
|  | Strict | *ermB* | macrolide, lincosamide, streptogramin, streptogramin A, streptogramin B | antibiotic target alteration | 98.78 | 98.79 |
|  | Strict | *efrA* | macrolide, fluoroquinolone, rifamycin | antibiotic efflux | 99.30 | 99.48 |
|  | Strict | *lnuB* | lincosamide | antibiotic inactivation | 99.63 | 100.00 |
|  | Strict | ANT(6)-Ia | aminoglycoside | antibiotic inactivation | 100.00 | 87.11 |
| #6933 | Strict | *dfrE* | diaminopyrimidine | antibiotic target replacement | 98.78 | 100.00 |
|  | Strict | *vanT* gene in *vanG* cluster | glycopeptide | antibiotic target alteration | 34.78 | 52.11 |
|  | Strict | *efrA* | macrolide, fluoroquinolone, rifamycin | antibiotic efflux | 99.65 | 99.48 |
| #7186 | Perfect | *dfrG* | diaminopyrimidine | antibiotic target replacement | 100.00 | 100.00 |
|  | Strict | *dfrE* | diaminopyrimidine | antibiotic target replacement | 98.78 | 100.00 |
|  | Strict | *tet*(M) | tetracycline | antibiotic target protection | 93.90 | 100.00 |
|  | Strict | *vanT* gene in *vanG* cluster | glycopeptide | antibiotic target alteration | 34.78 | 52.11 |
|  | Strict | *efrA* | macrolide, fluoroquinolone, rifamycin | antibiotic efflux | 99.30 | 99.48 |
| #5864 | Strict | *vanY* gene in *vanB* cluster | glycopeptide | antibiotic target alteration | 34.48 | 102.24 |
|  | Strict | AAC(6')-Ii | aminoglycosides | antibiotic inactivation | 99.45 | 100.00 |

**Supplementary Table S5 Proteomics analysis results of fHoEfm07 particle.** Proteins originating from the fHoEfm07 genome were identified from LC-MS/MS analysis conducted by the Proteomics Unit (University of Helsinki, Helsinki, Finland).

| **Protein Name** | **Locus Tag^1^** | **Proteotypic sequences (n)^2^** |
| --- | --- | --- |
| Portal protein | fHoEfm07_00003 | 10 |
| Head morphogenesis | fHoEfm07_00004 | 4 |
| LEMA protein | fHoEfm07_00006 | 5 |
| Hypothetical protein | fHoEfm07_00007 | 3 |
| Major head protein | fHoEfm07_00008 | 12 |
| DNA binding domain protein | fHoEfm07_00015 | 12 |
| Major capsid | fHoEfm07_00016 | 7 |
| Tail length tape measure protein | fHoEfm07_00018 | 33 |
| Distal tail protein Dit | fHoEfm07_00019 | 10 |
| Tail fiber protein; host specificity | fHoEfm07_00020 | 7 |
| Structural protein | fHoEfm07_00021 | 33 |
| Erf-like ssDNA annealing protein | fHoEfm07_00029 | 9 |

^1^ GenBank annotation table locus tag

^2^ Cut-off value 3 for proteotypic sequences was used for protein identification
